# Independent component analysis (ICA) applied to dynamic oxygen‐enhanced MRI (OE‐MRI) for robust functional lung imaging at 3 T

**DOI:** 10.1002/mrm.29912

**Published:** 2023-11-20

**Authors:** Sarah H. Needleman, Mina Kim, Jamie R. McClelland, Josephine H. Naish, Marta Tibiletti, James P. B. O'Connor, Geoff J. M. Parker

**Affiliations:** ^1^ Centre for Medical Image Computing (CMIC), Department of Medical Physics and Biomedical Engineering University College London London UK; ^2^ Wellcome/EPSRC Centre for Interventional and Surgical Sciences (WEISS), Department of Medical Physics and Biomedical Engineering University College London London UK; ^3^ Bioxydyn Limited Manchester UK; ^4^ BHF Manchester Centre for Heart and Lung Magnetic Resonance Research (MCMR), Manchester University NHS Foundation Trust Manchester UK; ^5^ Division of Radiotherapy and Imaging Institute of Cancer Research London UK

**Keywords:** dynamic, lung function, oxygenation, oxygen‐enhanced MRI, repeatability, susceptibility contrast

## Abstract

**Purpose:**

Dynamic lung oxygen‐enhanced MRI (OE‐MRI) is challenging due to the presence of confounding signals and poor signal‐to‐noise ratio, particularly at 3 T. We have created a robust pipeline utilizing independent component analysis (ICA) to automatically extract the oxygen‐induced signal change from confounding factors to improve the accuracy and sensitivity of lung OE‐MRI.

**Methods:**

Dynamic OE‐MRI was performed on healthy participants using a dual‐echo multi‐slice spoiled gradient echo sequence at 3 T and cyclical gas delivery. ICA was applied to each echo within a thoracic mask. The ICA component relating to the oxygen‐enhancement signal was automatically identified using correlation analysis. The oxygen‐enhancement component was reconstructed, and the percentage signal enhancement (PSE) was calculated. The lung PSE of current smokers was compared with nonsmokers; scan–rescan repeatability, ICA pipeline repeatability, and reproducibility between two vendors were assessed.

**Results:**

ICA successfully extracted a consistent oxygen‐enhancement component for all participants. Lung tissue and oxygenated blood displayed the opposite oxygen‐induced signal enhancements. A significant difference in PSE was observed between the lungs of current smokers and nonsmokers. The scan–rescan repeatability and the ICA pipeline repeatability were good.

**Conclusion:**

The developed pipeline demonstrated sensitivity to the signal enhancements of the lung tissue and oxygenated blood at 3 T. The difference in lung PSE between current smokers and nonsmokers indicates a likely sensitivity to lung function alterations that may be seen in mild pathology, supporting future use of our methods in patient studies.

## 1 INTRODUCTION

Dynamic oxygen‐enhanced MRI (OE‐MRI) is a functional imaging technique that utilizes inhaled oxygen as a contrast agent.^1^, ^2^ When applied to the lung, observation of the spatial distribution and temporal evolution of the OE‐MRI signal change can provide information relating to the delivery and uptake of oxygen.^3^ However, analysis of dynamic lung OE‐MRI is challenging due to the presence of confounds including artifacts and proton density changes arising from cardiac and respiratory motion, artifacts due to blood flow, and poor signal‐to‐noise ratio of lung tissue resulting from the extremely short parenchymal T_2_^*^ and its low proton density. The impact of these confounds masks the small amplitude oxygen‐induced signal change, which can reduce the sensitivity and accuracy of lung OE‐MRI.

Most lung OE‐MRI studies have focused on measuring T_1_‐related signal enhancements at 1.5 T. Because 3 T scanners are routinely used in clinical settings, development of lung OE‐MRI at this field strength is important to enable future clinical translation. However, as the MR field strength is increased, the T_2_^*^ dephasing increases^4^ and the longitudinal relaxivity of oxygen decreases.^5^ These can result in a greater masking of the underlying OE‐MRI signal by confounding factors and can reduce the accuracy and T_1_ sensitivity at 3 T relative to 1.5 T. Due to the greater impact of the confounding factors on lung OE‐MRI at 3 T, it is particularly attractive to develop approaches to separate the oxygen‐induced signal from confounds at this field strength.

Independent component analysis (ICA) is a data‐driven blind source separation technique for extracting different signal sources from measured data. The extracted signal sources, known as ICA components, linearly combine to form the measured data. The ICA components are assumed to be independent of each other.^6^, ^7^ Isolation of the oxygen‐enhancement signal response using ICA was demonstrated by Moosvi et al. in preclinical tumors.^8^ In this paper, we employ ICA to extract the oxygen‐enhancement signal from confounds to improve the sensitivity of OE‐MRI to alterations in lung function. We demonstrate the method in an experiment comparing the oxygen‐induced signal changes seen in healthy smokers and nonsmokers. The scan–rescan repeatability, the ICA pipeline repeatability, and the multi‐site multi‐vendor reproducibility of the technique are investigated.

## 2 THEORY

**FIGURE 1:**
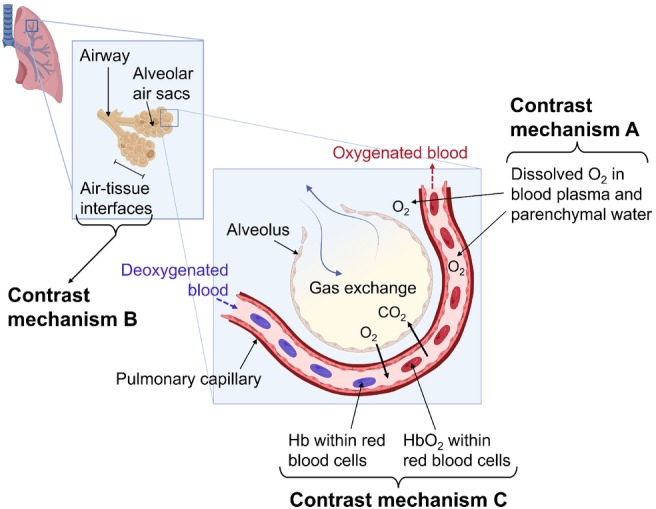
Diagram illustrating the main oxygen‐induced contrast mechanisms of the lung. Contrast mechanism A: During air inhalation, the blood is near full O_2_ saturation in healthy participants. During 100% O_2_ inhalation, excess O_2_ dissolves in blood plasma and lung tissue water. Dissolved O_2_ is paramagnetic, which can shorten T_1_. Hence, the increased concentration of dissolved O_2_ during 100% O_2_ inhalation causes T_1_ shortening of lung tissue and oxygenated blood. Contrast mechanism B: Magnetic susceptibility gradients arise due to gas–tissue interfaces in the lung, which can shorten T_2_^*^. Gaseous O_2_ has a greater magnetic susceptibility than air. As a consequence, 100% O_2_ inhalation increases the lung susceptibility gradients and causes T_2_^*^ shortening of lung tissue. Contrast mechanism C: Deoxygenated Hb molecules contained within red blood cells are paramagnetic. The paramagnetic Hb molecules generate magnetic susceptibility distortions, which can shorten T_2_^*^. In the blood, O_2_ binds to Hb molecules to form HbO_2_ molecules. However, Hb molecules in the blood are close to full O_2_ saturation during air inhalation. 100% O_2_ inhalation therefore results in a minor increase in blood oxygen saturation. The Hb concentration decreases, causing the T_2_^*^ of oxygenated blood to increase (BOLD contrast). Created with BioRender.com. Hb, hemoglobin; HbO_2_, oxyhemoglobin; O_2_, oxygen.

Several different MR contrast mechanisms are induced in lung OE‐MRI, which contribute to the measured oxygen‐enhancement response. During the inhalation of pure oxygen, excess molecular oxygen dissolves in lung tissue water and oxygenated pulmonary capillaries and veins. Molecular oxygen is paramagnetic; hence, an increased concentration of dissolved oxygen causes T_1_ shortening in lung tissue and oxygenated blood^1^ (illustrated by contrast mechanism A in Figure 1).

Figure 1 also illustrates the two major T_2_^*^ contrast mechanisms in the lung. Represented by contrast mechanism B, T_2_^*^ can be affected by changes in magnetic susceptibility arising due to gas–tissue interfaces in the lung. Because gaseous oxygen has a greater magnetic susceptibility than air, the inhalation of pure oxygen increases the susceptibility gradients within the lung and causes T_2_^*^ shortening.^9^, ^10^

Contrast mechanism C demonstrates the effect of deoxyhemoglobin on T_2_^*^ Deoxyhemoglobin is paramagnetic and creates local magnetic field distortions that induce T_2_^*^ shortening (i.e., BOLD contrast).^11^ Oxygenated pulmonary blood is close to full saturation during air inhalation for healthy participants.^12^ Consequently, the inhalation of pure oxygen results in a minimal change to the blood oxygen saturation, producing a small BOLD effect.^13^

## 3 METHODS

### 3.1 Single center OE‐MRI acquisition

Twenty‐three healthy participants were imaged on a 3 T Philips Ingenia MRI scanner (Philips Healthcare, Best, Netherlands) after local institutional review board approval (18837/001, UCL Research Ethics Committee) and written informed consent. The recruited participants had no previous record of lung disease; participant information is summarized in Table 1A (details of the nonsmokers and current smokers involved in the study is provided in Table S1). The dynamic OE‐MRI protocol used a 2D coronal multi‐slice dual‐echo T1‐fast field echo (RF‐spoiled gradient echo) sequence to acquire four posterior slices during free‐breathing.^14^ TEs of TE_1_/TE_2_ = 0.72/1.2 ms were used; full sequence details are provided in Table S2A.

**TABLE 1:** Summary of participant information for (A) the healthy participant study, (B) the repeatability study, and (C) the reproducibility study.

		(A)	(B)	(C)
Participants	Total	23	8	8
	Male	13	4	3
	Female	10	4	5
Age (years)	Mean	34	30	40
	Range	22–55	23–51	26–54
Smoking status	Currently smoke regularly	5	0	0
Pack years	Mean	2.3	0	1.5
	Range	0–14	0	0–8

*Note*: All recruited participants were healthy and had no previous record of lung disease. Some participants were common across groups. Further details of the nonsmokers and current smokers involved in the healthy participant study (A) are provided in Table S1.

Participants inhaled medical air (21% oxygen [O_2_]) and pure oxygen (100% O_2_) during the dynamic scan; gas was delivered via a non‐rebreathing face mask (Intersurgical Ltd., Wokingham, UK) at a flow rate of 15 L min^−1^. The delivered gas was cycled between air and 100% O_2_ three times, as depicted in Figure S1. The cyclic gas delivery imposed a temporal modulation on the oxygen‐enhancement MR signal, designed to increase ICA sensitivity^8^ and aid identification of the ICA component relating to the oxygen‐enhancement response. The delivered gas was manually switched between air and 100% O_2_ every 1.5 min using a gas blender (IHC Low Flow Blender, Inspiration Healthcare Ltd., Earl Shilton, UK). The 1.5‐min gas period was chosen to minimize the total dynamic scan duration, thereby maximizing participant scan tolerability in future patient studies.

### 3.2 Data postprocessing

Motion correction of the dynamic images was performed using the deformable image registration software NiftyReg (version 1.5.71).^15^ The registration parameters implemented in NiftyReg are provided in Table S3. The 2D registration aligned all dynamic images with a reference image, processed separately for each slice. The reference image was chosen to represent the mean lung position and was identified as the dynamic image with the greatest correlation to the average of all the images in the dynamic series. The reference images were manually segmented by intensity thresholding in ImageJ (version 1.52a).^16^ Two anatomical masks were created (shown in Figure S2): a mask of lung tissue excluding major vasculature, and a mask of the full thoracic cavity including the heart and major vessels. No spatial smoothing was applied to the data.

### 3.3 Application of ICA

Temporal ICA was applied to the thoracic masked dynamic OE‐MRI time series using scikit‐learn (version 1.2.2) FastICA^17^, ^18^ separately for each echo. The number of ICA components separated during the analysis can affect the form of the resulting components. However, determination of the optimum number of components to separate is an unsolved problem, and the optimum number of components may vary between subjects. Additionally, the ordering of the ICA components is arbitrary. Therefore, it was necessary to identify the ICA component relating to the oxygen‐enhancement signal (referred to as the *OE ICA component*) and the number of ICA components to use for each dataset.

ICA was repeatedly run using an increasing number of components from 22 to 72 (51 separate instances) for each dataset. Initial experiments showed that this range of component numbers enabled the OE ICA component to be reliably identified, and use of fewer or more components did not improve the ability of ICA to extract the OE ICA component. The Spearman correlation coefficient of a sinusoidal approximation of the oxygen‐induced signal (Figure S3) to every component from all runs of ICA was calculated using SciPy (version 1.8.1).^19^ The correlation values were compared across all ICA components, and the single component with the greatest correlation value was identified as the optimal OE ICA component for the dataset under consideration (illustrated in Figure S3). Hence, the ICA pipeline enables the automatic identification of the optimal OE ICA component and overcomes the ambiguity of the number of ICA components to use.

If the magnitude of the correlation coefficient of the identified optimal OE ICA component was less than 0.4, the ICA application process was repeated for the dataset under consideration. A correlation value of less than 0.4 indicated the extraction of a poor OE ICA component, likely due to the ICA algorithm getting stuck in a bad local optimum. Known as *algorithmic uncertainty*, the convergence of ICA at a local optimum, rather than the global optimum, may occur due to the random initialization of ICA.^20^, ^21^ The algorithmic uncertainty of ICA within the pipeline was investigated and is described later.

The MR signal originating from the OE ICA component was reconstructed for comparison with the raw motion‐corrected MRI data. The percentage signal enhancement (PSE) of the reconstructed OE ICA component data (PSE_ICA_) and the raw motion‐corrected MRI data (PSE_MRI_) were calculated. The PSE describes the MR signal change that occurred upon the inhalation of pure oxygen and can be used to map the oxygen‐induced signal response across the lung.

The PSE time series were calculated using Equation (1), where SI_air_ is the mean air‐inhalation MRI signal intensity (SI) image, and SI(*t*) is the dynamic image at time *t*. SI_air_ was calculated by averaging the first 60 dynamic images. PSE maps were generated using the mean air‐inhalation image, SI_air_, and the mean oxygen‐inhalation image, SI_oxy_, in Equation (2). SI_oxy_ was calculated by averaging the final five dynamic images from each of the three oxygen‐inhalation periods (a total of 15 images). Hence, for the cyclic gas delivery scheme with 1.5‐min gas periods, the PSE maps describe the signal enhancement that occurred during 1.5 min of oxygen inhalation.

(1) $$\mathrm{PSE}(t)=\frac{\mathrm{SI}(t)-{\mathrm{SI}}_{\mathrm{air}}}{{\mathrm{SI}}_{\mathrm{air}}}\times 100\%$$

(2) $$\mathrm{PSE}=\frac{{\mathrm{SI}}_{\mathrm{oxy}}-{\mathrm{SI}}_{\mathrm{air}}}{{\mathrm{SI}}_{\mathrm{air}}}\times 100\%.$$

The median PSE_MRI_ and PSE_ICA_ map value within the lung mask was assessed for each subject. To probe the sensitivity of the OE ICA component to differences in the signal enhancements between slices, the median lung PSE_ICA_ was also calculated for each slice. The median lung PSE_ICA_ was compared between all permutations of slice pairs from nonsmoker participants using a paired test (sign test) due to outliers in the pairs of differences. The Bonferroni correction for multiple comparisons was applied; *p* < 0.008 was considered significant. All statistical analyses were performed using IBM SPSS Version 28.0 (SPSS Statistics for Windows, IBM Corp., Armonk, NY, USA).

The median lung PSE_MRI_ and PSE_ICA_ values were compared between nonsmoker and current smoker participants using an unpaired (independent samples) t‐test; *p* < 0.05 was considered significant. Multiple regression was employed to adjust for the confounds of age and gender on the smoking status comparison. Separate models were created for each set of data (both echoes of PSE_MRI_ and PSE_ICA_) containing current smoking status, age, and gender as variables.

### 3.4 Repeatability and reproducibility

Four‐to‐six week (mean 5 weeks) repeat scans of eight healthy participants were performed to assess the repeatability of the developed ICA OE‐MRI analysis technique. The scan–rescan repeatability study used the 3 T Philips Ingenia scanner with protocol settings as described above. A reproducibility study was also carried out: eight healthy participants were scanned on the same 3 T Philips Ingenia scanner and on a 3 T Siemens MAGNETOM Vida scanner (Siemens Healthineers, Erlangen, Germany) located in a different institution using a multi‐slice 2D double echo FLASH acquisition, within a 4‐6‐week interval (mean 5 weeks). Due to sequence implementation differences, TEs were increased to TE_1_/TE_2_ = 0.81/1.51 ms for the Siemens scan (further details of the Siemens MAGNETOM Vida implementation are provided in Table S2B). Participant information for the scan–rescan and reproducibility studies are summarized in Table 1B, C, respectively. One scan–rescan participant was excluded from the analysis due to the presence of substantial diaphragm ghosts in the second scan.

The repeatability and reproducibility scans were processed and analyzed using ICA as described above. For the scan–rescan repeatability study, the median lung PSE_ICA_ was compared between the repeat scans of each echo using Bland–Altman analysis, including bias and limits of agreement, the repeatability coefficient, and two‐way single measure mixed‐effects model intraclass correlation coefficient (ICC) with absolute agreement.^22^, ^23^ Identical comparisons were made for the interquartile range (IQR) of the lung PSE_ICA_ between repeat scans. The ICC values were taken to indicate moderate repeatability if between 0.5–0.75, good repeatability if between 0.75−0.9, and excellent repeatability if greater than 0.9.^24^ Variations in the measured PSE between the Siemens scan and the Philips scan were expected due to the different TEs implemented (predicted by sequence‐specific signal simulations^14^, ^25^ shown in Figure S4). As a result, we were unable to perform direct comparisons of PSE_ICA_ for the reproducibility study. Bland–Altman analysis was used to compare the TE trend of the reproducibility study PSE_ICA_ to the signal simulations.

The repeatability of ICA within the pipeline was separately examined to assess algorithmic uncertainty by a repeat application of the pipeline to the scan–rescan participants. As for the scan–rescan study, Bland–Altman analysis was performed and the repeatability coefficient and ICC were calculated to compare the median lung PSE_ICA_ and the PSE_ICA_ IQR between repeat pipeline applications.

## 4 RESULTS

### 4.1 Extraction of the oxygen‐enhancement signal response

**FIGURE 2:**
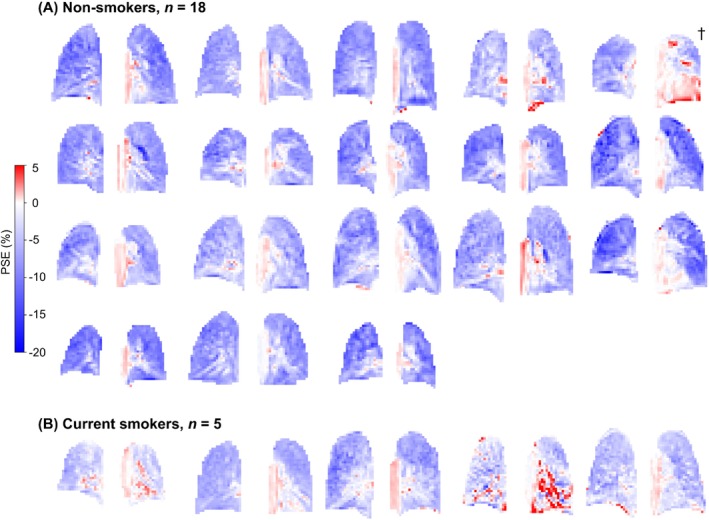
PSE_ICA_ maps of (A) nonsmoker participants and (B) current smoker participants, all echo 1 data. The slice containing the aorta is shown for each participant; cardiac tissue is present in the lower left lung of the nonsmoker labeled †. Minor variations between PSE_ICA_ maps arose due to differences in blood vessel and cardiac tissue content of the slice. An asymmetric color bar range of (−20, 5) is used to show the negative enhancement of the lung tissue and the positive enhancement of the heart and aorta (see also simulations in Figure S4). ICA, independent component analysis; PSE, percentage signal enhancement.

The ICA analysis pipeline successfully extracted and identified the optimal OE ICA component from both echoes of every participant. The cyclic OE‐MRI protocol was tolerated well by all participants and no adverse events were recorded. Across the participants, the optimal OE ICA component was found in runs of ICA in which different numbers of ICA components were used: range of 22–53 components (mean 30) for echo 1, and range of 22–52 components (mean 30) for echo 2. The nonsmoker PSE_ICA_ maps demonstrated consistent signal distributions within the lung (each subject is presented in Figure 2A) with minor variations between maps due to the slice location, blood vessel content, and presence of cardiac tissue. The PSE_ICA_ time series of all nonsmokers displayed a cyclical signal enhancement in response to the air–oxygen gas switching (each subject is presented in Figure S5A).

**FIGURE 3:**
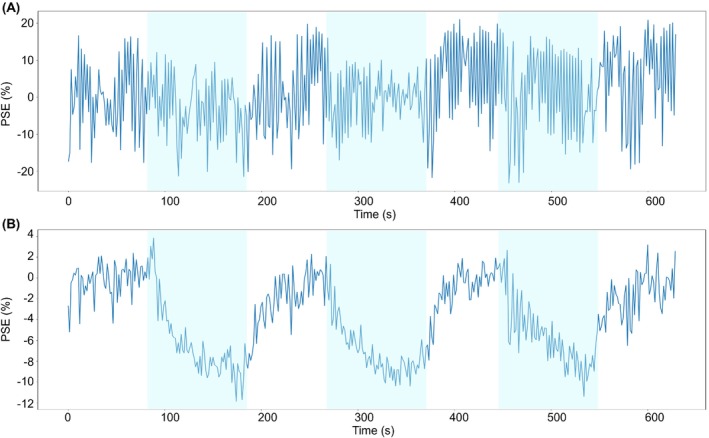
Time series of the median PSE within the lung mask of (A) PSE_MRI_ data and (B) PSE_ICA_ data, both echo 1. Shaded blue periods indicate pure oxygen inhalation. ICA, independent component analysis; PSE, percentage signal enhancement.

Example ICA components extracted from each echo of a nonsmoking participant are presented in Figures S6 and S7. For this participant, the Spearman correlation method identified the optimal OE ICA component in the run of ICA using 22 components for echo 1 and in the run of ICA using 23 components for echo 2. Figure 3 presents the median echo 1 PSE time series within the lung mask for (A) PSE_MRI_ and (B) PSE_ICA_ of the same nonsmoking participant; frequency spectra of the PSE time series are shown in Figure S8. The PSE_ICA_ time series exhibited well‐defined cyclic enhancement compared to the PSE_MRI_ time series. The PSE_ICA_ frequency spectrum contained a sharp peak at the gas cycling frequency and minimal amplitudes at higher frequencies, whereas the amplitude of the gas cycling frequency peak was lower in the PSE_MRI_ spectrum (spectra shown normalized by the maximum amplitude), and the PSE_MRI_ spectrum contained substantial frequency amplitudes within the cardiac and respiratory ranges.

**FIGURE 4:**
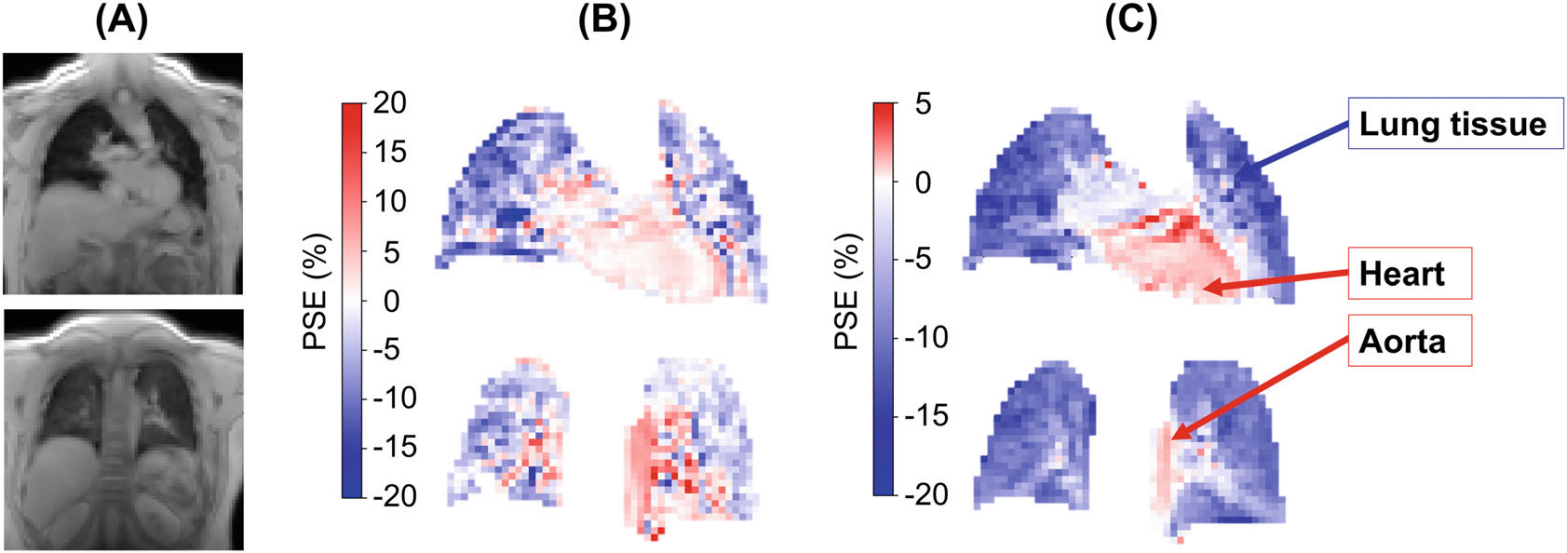
PSE maps of the nonsmoking participant in Figure 3, all echo 1 data within the thoracic mask. (A) MRI images for anatomical reference; (B) PSE_MRI_ maps; and (C) PSE_ICA_ maps. For both the PSE_MRI_ and PSE_ICA_, the signal enhancement of the lung tissue occurred with a negative PSE, whereas the heart and aorta had a positive PSE. The PSE maps are presented using different color bar ranges to illustrate the different magnitudes of positive PSE. An asymmetric color bar range of (−20, 5) is used for the PSE_ICA_ maps (C) to show the negative enhancement of the lung tissue and the small positive enhancement of the heart and aorta (see also simulations in Figure S4), whereas a symmetric color bar range of (−20, 20) is used for the PSE_MRI_ maps to display the regions of strong positive signal enhancement within the lung that were not observed in the PSE_ICA_ maps. ICA, independent component analysis; PSE, percentage signal enhancement.

For both PSE_MRI_ and PSE_ICA_, the signal enhancement of the lung tissue occurred with a negative PSE, whereas the heart and aorta had a positive PSE, as demonstrated by the PSE_ICA_ maps in Figure 4C. Regions of strong positive signal enhancement within the lung were observed in the PSE_MRI_ maps (Figure 4B), which were not seen in the PSE_ICA_ maps. The low amplitude opposite enhancements of the lung tissue from oxygenated blood contained within the heart and aorta were predicted by sequence‐specific signal simulations^14^, ^25^ (Figure S4). Additionally, the simulations predicted a negative lung PSE at TEs greater than 0.23 ms due to the dominance of ΔT_2_^*^ effects—a negative lung PSE was observed by our experiment using TEs of 0.71 and 1.2 ms. For TEs shorter than 0.23 ms, the simulations predicted a small positive lung PSE due to the dominance of ΔT_1_ effects.

Figure S9 presents the median lung PSE_ICA_ measured in each slice of the nonsmoking participants. For echo 1, there is a visible trend for the magnitude of negative lung PSE_ICA_ to be reduced in posterior slices, whereas for echo 2 the magnitude of negative lung PSE_ICA_ appears to be greater in posterior slices. For echo 2, the median lung PSE_ICA_ of slice 3 was significantly different than both slice 1 and slice 2 (*p* = 0.008 for slice 1; *p* = 0.001 for slice 2) (Table S4).

### 4.2 Smoking status PSE comparison

**FIGURE 5:**
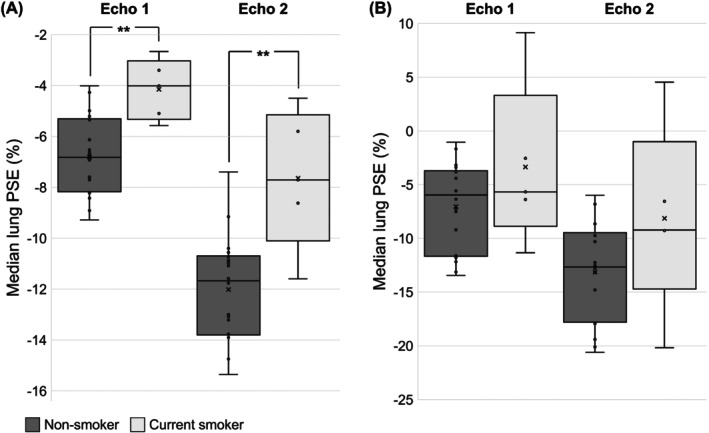
Comparison between the median lung PSE map value of nonsmoker (gray) and current smoker (white) participants for each echo of (A) the PSE_ICA_ data and (B) the PSE_MRI_ data, without adjustment for the confounds of age and gender. The PSE_ICA_ of current smokers was significantly smaller than nonsmokers for both echoes: *p* = 0.002 for echo 1; *p* < 0.001 for echo 2. No significant difference was present in the PSE_MRI_ data: *p* = 0.154 for echo 1; *p* = 0.091 for echo 2. ICA, independent component analysis; PSE, percentage signal enhancement.

The PSE_ICA_ maps of all current smoker participants are displayed alongside those of nonsmoker participants in Figure 2, with corresponding median lung PSE_ICA_ time series shown in Figure S5. Figure 5 presents a comparison between the median lung PSE of current smoker and nonsmoker participants for both PSE_MRI_ and PSE_ICA_. The median lung PSE_ICA_ was significantly smaller in current smokers than nonsmokers: *p* = 0.002 for echo 1 and *p* < 0.001 for echo 2, whereas no significant difference was observed for the median lung PSE_MRI_: *p* = 0.154 for echo 1 and *p* = 0.091 for echo 2.

Table S5 contains a summary of the multiple regression results adjusted for the effects of age and gender. After adjustment, current smoking status remained significant for both echoes of the PSE_ICA_ data (*p* < 0.050 for echo 1; *p* = 0.033 for echo 2). In addition, gender was significant for PSE_ICA_ echo 2 (*p* = 0.043). For the PSE_MRI_ data, current smoking status remained nonsignificant after adjustment. Age was significant for both echoes of the PSE_MRI_ data (*p* = 0.034 for echo 1; *p* = 0.031 for echo 2).

### 4.3 Scan–rescan repeatability, ICA pipeline repeatability, and multi‐site reproducibility

**FIGURE 6:**
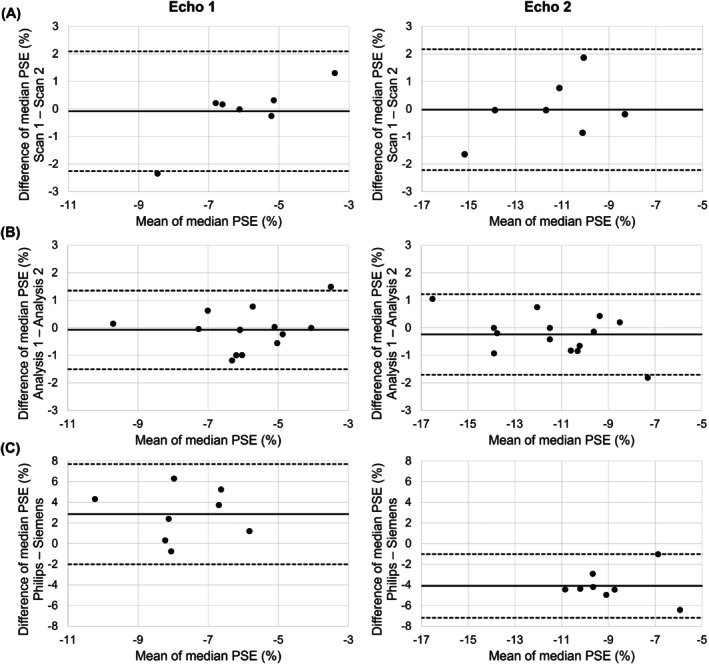
Bland–Altman plots of the median lung PSE_ICA_ values from the analysis of: (A) the scan–rescan repeatability; (B) the repeatability of ICA within the pipeline; and (C) the multi‐site reproducibility. The solid black line indicates the bias, and the dashed black lines indicate the limits of agreement. Bland–Altman analysis results are presented in Table S6. ICA, independent component analysis; PSE, percentage signal enhancement.

**FIGURE 7:**
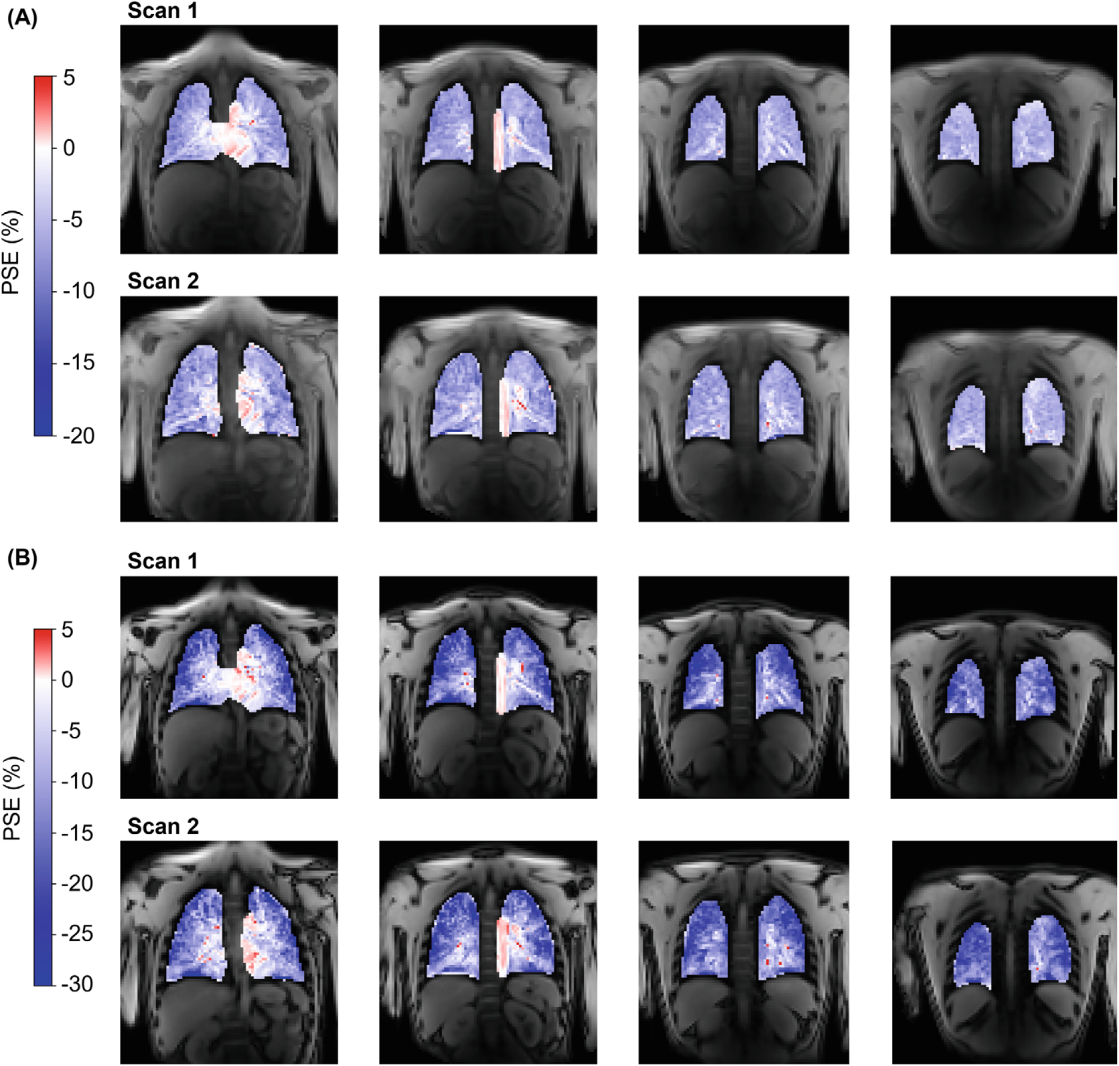
Example scan–rescan PSE_ICA_ maps for a nonsmoker repeatability study participant. (A) Echo 1 data from both scans; (B) echo 2 data from both scans. The lung PSE_ICA_ from echo 2 was more negative than echo 1 for both scans. Asymmetric color bar ranges of (−20, 5) for echo 1 and (−30, 5) for echo 2 are used to show the negative enhancement of the lung tissue and the positive enhancement of the heart and aorta (see also simulations in Figure S4). ICA, independent component analysis; PSE, percentage signal enhancement.

The scan–rescan repeatability, ICA pipeline repeatability, and multi‐site reproducibility results are presented in Figure 6 (Bland–Altman plots of the median lung PSE_ICA_), Figure S12 (Bland–Altman plots of the IQR of the lung PSE_ICA_), and Table S6 (statistical analysis). The repeatability analysis was presented in part at the joint annual meeting of the International Society for Magnetic Resonance in Medicine and the International Society for Magnetic Resonance Radiographers and Technologists, London, 2023.^26^ Example scan–rescan PSE_ICA_ maps are displayed in Figure 7 for a nonsmoker participant, in which similar spatial distributions of PSE_ICA_ were observed between scans. The ICC values indicated good repeatability for both echoes: 0.807 for echo 1 and 0.907 for echo 2. No significant biases were present in the median lung PSE_ICA_ value between scans: −0.085% for echo 1 and −0.023% for echo 2.

**FIGURE 8:**
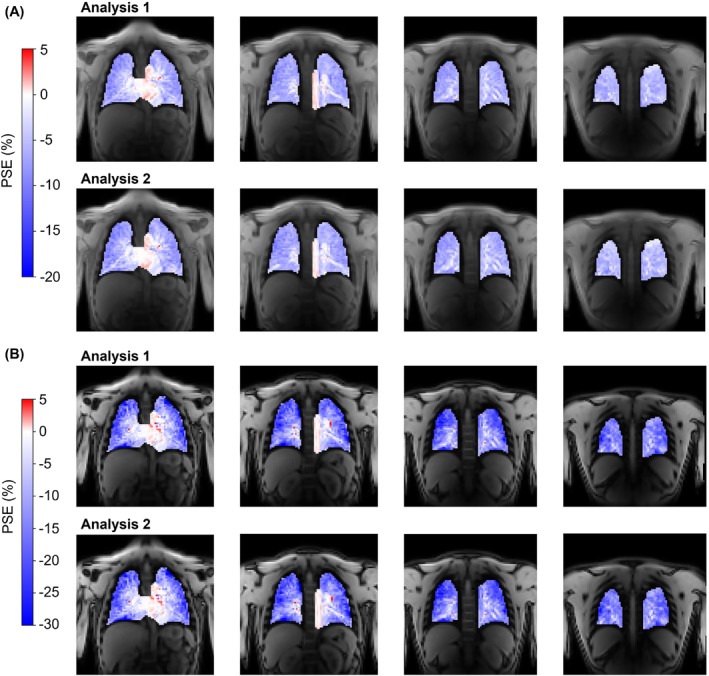
Example PSE_ICA_ maps produced by the repeat application of the pipeline for the nonsmoker participant shown in Figure 7 for (A) echo 1 data and (B) echo 2 data. (Figure 7 presents the PSE_ICA_ scan–rescan maps; the current figure presents the PSE_ICA_ maps from the repeat pipeline application to data from scan 1.) The repeat pipeline application to echo 1 data produced similar PSE_ICA_ maps for which structures within the lung were consistent. Identical results were produced by the repeated pipeline application to echo 2 data. Asymmetric color bar ranges of (−20, 5) for echo 1 and (−30, 5) for echo 2 are used to show the negative enhancement of the lung tissue and the positive enhancement of the heart and aorta (see also simulations in Figure S4). ICA, independent component analysis; PSE, percentage signal enhancement.

Example PSE_ICA_ maps produced by the repeat application of the pipeline are displayed in Figure 8 for a nonsmoker participant. Structures within the lung appeared consistent in the PSE_ICA_ maps from the repeated pipeline application to echo 1 data. Identical OE ICA components were extracted by the repeat pipeline application to echo 2 data. The repeat application of the ICA analysis pipeline displayed better repeatability than the scan–rescan analysis, demonstrated by the excellent ICC values of 0.926 for echo 1 and 0.958 for echo 2. No significant biases were observed: −0.075% for echo 1 and −0.240% for echo 2.

The ICC values of the IQR of the scan–rescan PSE_ICA_ indicated moderate repeatability (0.669 for echo 1; 0.525 for echo 2), whereas the ICC values of the IQR of the repeat pipeline application indicated excellent repeatability (0.971 for echo 1; 0.942 for echo 2). The ICC values for the IQR of the PSE_ICA_ were greater for echo 1 than echo 2 for both the scan–rescan repeatability and the repeat pipeline application. For the median PSE_ICA_ comparison, the ICC values were greater for echo 2 than echo 1 for both the scan–rescan repeatability and the repeat pipeline application. No significant biases were present in the IQR of the PSE_ICA_ for both the scan–rescan repeatability (0.558% for echo 1; 0.685% for echo 2) and for the repeat application of the pipeline (−0.009% for echo 1; 0.156% for echo 2).

**FIGURE 9:**
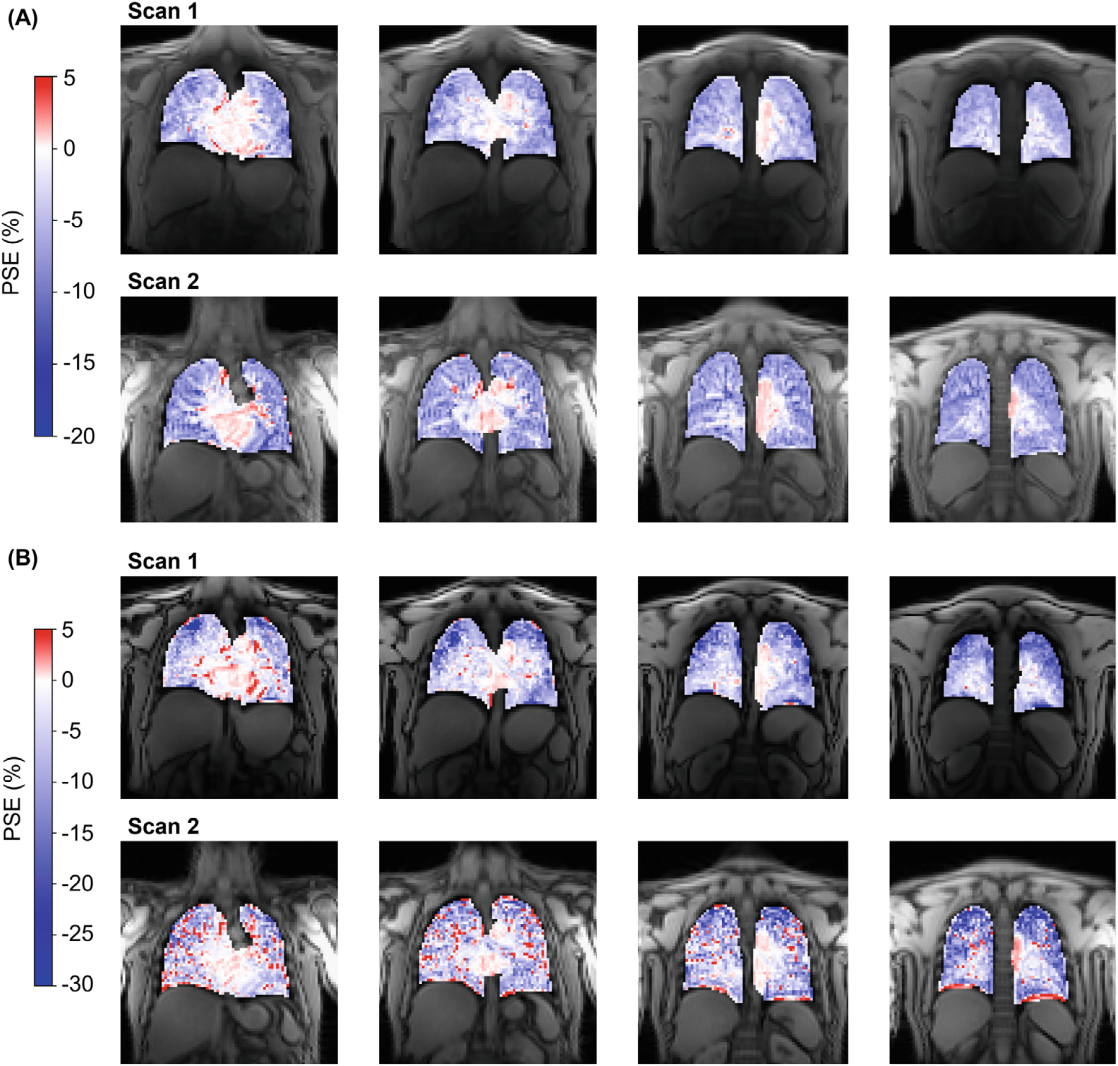
Example PSE_ICA_ maps for a nonsmoker reproducibility study participant. (A) Echo 1 data from both scans; (B) echo 2 data from both scans. Scan 1 was performed on a Philips Ingenia (Philips Healthcare, Best, Netherlands) (TE_1_/TE_2_ = 0.71/1.2 ms), and scan 2 was performed on a Siemens MAGNETOM Vida (Siemens Healthineers, Erlangen, Germany) (TE_1_/TE_2_ = 0.81/1.51 ms). Asymmetric color bar ranges of (−20, 5) for echo 1 and (−30, 5) for echo 2 are used to show the negative enhancement of the lung tissue and the positive enhancement of the heart and aorta (see also simulations in Figure S4). ICA, independent component analysis; PSE, percentage signal enhancement.

Figure 9 presents the PSE_ICA_ maps from both scans of a nonsmoker reproducibility study participant. Similar spatial patterns of PSE_ICA_ were seen between the Siemens scan and the Philips scan; however, the Siemens scan echo 2 PSE_ICA_ maps generally appeared to contain substantial noise‐like patterns of positive PSE_ICA_ within the lung. Minor biases of 2.853% for echo 1 and −4.095% for echo 2 were observed. The signal simulation predicted a more negative PSE at each TE of the Siemens scan than the corresponding TE of the Philips scan, which would give rise to a positive bias between the two systems (Figure S4B). The predicted positive bias was observed for echo 1. However, contrary to the signal simulations, a negative bias was observed for echo 2 as the Siemens scan PSE_ICA_ was less negative than the Philips scan PSE_ICA_.

## 5 DISCUSSION

A previous study reported the use of ICA to extract the oxygen‐induced signal change in preclinical tumor models.^8^ However, there are no published studies demonstrating the use of ICA to extract the oxygen‐induced signal change in the human lung. To the best of our knowledge, we present the first application of ICA to lung OE‐MRI. We developed an automatic analysis pipeline, utilizing ICA and a cyclic gas challenge, to extract the oxygen‐induced signal change from the confounding factors present in lung MRI. The developed pipeline orders the ICA components using cross‐correlation to identify the “optimal” oxygen‐enhancement ICA component for each dataset. The objective and automatic approach for identifying the oxygen‐enhancement signal increases the robustness of the pipeline and overcomes the main ambiguities of ICA in the ordering of components and in the number of components to use. We also show that our OE‐MRI acquisition and ICA analysis approach can be applied at 3 T to exploit the largely T_2_^*^‐driven contrast observed at this field strength. We have published the developed ICA pipeline code on GitHub: https://github.com/SarahNeedleman/OE‐MRI_ICA_2023.

### 5.1 Sensitivity to smoking status

The significant difference between the median lung PSE_ICA_ of current smokers and nonsmokers, which remained after adjustment for the effects of age and gender, suggests that our OE‐MRI acquisition and analysis method is sensitive to smoking‐related changes of the lung. In contrast, no significant difference was observed between the median lung PSE_MRI_ of current smokers and nonsmokers, both with and without adjustment for age and gender. The lack of sensitivity to smoking status by the raw motion‐corrected MRI data may be due to the presence of noise and confounding factors in the lung MRI data, which were reduced by ICA in the OE ICA component.

Age was significant for both echoes of the PSE_MRI_ data but not for the PSE_ICA_ data. Age‐related anatomical or physiological changes may have altered the MR signal, the effects of which ICA was able to reduce in the OE ICA component. However, the presented smoking status comparison is limited by the small number of participants in the study and by the gender imbalance—all current smoker participants were male. A larger study with balanced groups may provide further insights into the effects of age and gender on the oxygen‐induced signal response of the lung.

### 5.2 Scan–rescan repeatability, ICA pipeline repeatability, and multi‐site reproducibility

No significant PSE_ICA_ biases were observed for the scan–rescan study or for the repeat application of the pipeline to the scan–rescan data. The limits of agreement of the median lung PSE_ICA_ from the repeat application of the pipeline were smaller than, but of the same magnitude as, the scan–rescan study. This suggests that the algorithmic uncertainty of ICA contributes considerably to the overall variance of the OE‐MRI experiment. The remaining variance in the scan–rescan study may be due to physiological variation including respiratory motion and motion‐induced density changes, subject positioning, subject motion, and noise.

The ICC values of the IQR of the lung PSE_ICA_ for the scan–rescan repeatability were lower than those from the repeat application of the pipeline. It is likely that variation in the subject positioning of the repeat scans resulted in a different blood vessel content of the slices compared. Differences in the blood vessel content of the compared slices were expected to have substantially impacted the measured PSE_ICA_ IQR due to the positive PSE contribution from oxygenated blood vessels. Partial volume effects of oxygenated blood vessels within the lung would act to reduce the magnitude of the measured negative lung PSE_ICA_ and to increase the PSE_ICA_ IQR. This likely reduced the scan–rescan repeatability of the PSE_ICA_ IQR relative to the repeat pipeline application. Overall, the scan–rescan median PSE_ICA_ ICC values indicate a good repeatability of the OE‐MRI ICA approach and suggest that the pipeline is suitable for cross‐sectional and longitudinal assessment.

When compared with the scan–rescan repeatability study, the reproducibility study biases were greater in magnitude but still less than 5% (Table S6). Greater biases were expected for the reproducibility study due to the different TEs achievable on the two MR systems used. A negative bias was observed for echo 2 data because the PSE_ICA_ from the Siemens scan was less negative than the PSE_ICA_ from the Philips scan, contrary to the signal simulations. The Siemens scan echo 2 PSE_ICA_ map, shown in Figure 9B (scan 2), contained pixels with close to zero, and positive, PSE_ICA_ in the lung tissue. It is possible that the signal from the second echo of the Siemens scan was noise limited, which resulted in the less negative lung PSE_ICA_. Overall, we have demonstrated feasibility of the OE‐MRI acquisition approach and analysis pipeline for use in multi‐center studies and on different MRI vendor scanners.

### 5.3 Oxygen‐induced MR contrast mechanisms

The lung tissue exhibited an opposite oxygen‐induced signal change relative to the small positive PSE seen in the heart and aorta, which contain oxygenated blood. The opposite signal responses arise due to the dominance of T_2_^*^ contrast in lung tissue and T_1_ contrast in oxygenated blood, as predicted by the signal simulations shown in Figure S4. The oxygen‐induced T_2_^*^ shortening of the lung, giving rise to a negative signal change, occurs due to an increase in the gas–tissue susceptibility gradients resulting from the elevated concentration of gaseous oxygen in the lung (contrast mechanism B in Figure 1).^9^

The oxygen‐induced T_1_ contrast mechanism is generated by an increased concentration of dissolved oxygen in lung tissue water and blood, which causes T_1_ shortening (contrast mechanism A in Figure 1). This T_1_ shortening, and resulting positive signal change, contributes to the measured lung PSE due to the inherent T_1_‐weighting in the spoiled gradient echo acquisition. However, as predicted by the signal simulations, the lung tissue signal change is dominated by ΔT_2_^*^ effects, instead of ΔT_1_ effects, at the TEs in our experiment, which gave rise to the negative PSE we observed in lung tissue.

For oxygenated blood in which gas–tissue susceptibility gradients do not exist, the dissolved oxygen‐induced T_1_ contrast is predicted to dominate the OE‐MRI signal change. This T_1_ contrast mechanism shortens the local T_1_ to generate a small positive PSE, as was observed in the aorta. The T_2_^*^ of oxygenated blood can be altered by susceptibility distortions generated by paramagnetic deoxyhemoglobin (BOLD contrast; mechanism C in Figure 1). The inhalation of pure oxygen can reduce the concentration of deoxyhemoglobin, causing T_2_^*^ to increase and generating a positive signal change. However, because oxygenated blood is almost fully saturated in healthy participants during air inhalation,^12^ the inhalation of pure oxygen results in a minimal change to the concentration of deoxyhemoglobin. Hence, it is anticipated that BOLD contributions to ΔT_2_^*^ and the PSE of oxygenated blood are minor.^9^, ^13^

As discussed above, the T_2_^*^‐sensitive OE‐MRI method we have developed at 3 T was sensitive to, and able to resolve, the different oxygen‐induced MR signal changes of lung tissue from oxygenated blood, which has not previously been observed using OE‐MRI. The majority of lung OE‐MRI studies have focused on the use of T_1_‐weighted sequences at 1.5 T^27^ due to the shorter T_2_^*^ of the lung and the reduced T_1_ relaxivity of oxygen^5^ at higher field strengths. Lung OE‐MRI studies reported at 3 T have also utilized T_1_‐weighted sequences, and as a result, did not exhibit sensitivity to the opposite T_2_^*^ and T_1_ signal changes of the lung and oxygenated blood.^28^, ^29^, ^30^ Hence, the use of T_2_^*^‐sensitive sequences is a promising direction for the use of OE‐MRI at 3 T for functional lung imaging.

The measured MR signal is influenced by changes in lung proton density, lung gas volume, and tissue geometry that occur during the respiratory cycle. Variation in the proton density of the lung affects the measured proton MR signal, which can compromise the accuracy and repeatability of lung OE‐MRI.^14^ To reduce the impact of tissue density fluctuations, and to improve repeatability of lung MR techniques, density‐induced signal changes of the lung are often corrected for prior to analysis. We chose not to apply a density correction to the free‐breathing dynamic OE‐MRI data input to the ICA pipeline for a number of reasons. Application of a density correction may introduce errors if the modeled relationship between the MR signal and lung volume is imprecise and unable to capture the complexity of lung motion. As a result, complexities such as local ventilation variations due to disease, irregular breathing, and large volume changes may not be fully corrected for. By avoiding the use of a density correction, we aimed to avoid introducing such errors into the analysis of the dynamic OE‐MRI data.

We speculated that ICA may be able to separate the density‐related signal fluctuations from the OE ICA component so that a density correction would not be required. Proton density‐induced changes to the MR signal intensity were likely to have contributed to the fluctuations observed in the PSE_MRI_ time series (Figure 3) and to have given rise to the substantial respiratory frequency amplitudes in the PSE_MRI_ frequency spectrum (Figure S8). In contrast, the PSE_ICA_ frequency spectrum contained low amplitudes within the respiratory frequency range suggesting minimal contamination of the OE ICA component by proton density changes. Hence, we conclude that the ICA pipeline does enable the analysis of dynamic free‐breathing lung OE‐MRI without the need to apply a density correction.

The measured MR signal can also be affected by the lung gas volume and tissue geometry. Changes in lung gas volume and tissue geometry alter T_2_^*^: lung T_2_^*^ is shorter at end‐inspiration, which decreases the MR signal intensity at this respiratory state.^4^, ^10^, ^31^ As for the proton density‐induced changes to the MR signal intensity, the respiratory motion‐induced changes to T_2_^*^ were likely to have contributed to the noise in the PSE_MRI_ time series (Figure 3) and to the substantial respiratory frequency amplitudes in the PSE_MRI_ frequency spectrum (Figure S8). Because the amplitudes of respiratory frequencies were minimal in the PSE_ICA_ spectrum, it is likely that ICA effectively removed signals relating to respiratory motion‐induced T_2_^*^ changes from the OE ICA component.

The reference image for NiftyReg registration was chosen as the image that most closely represented the mean lung position of the dynamic image series. However, because the reference image identification process was performed separately for each slice, the reference image of one slice may not correspond to the same dynamic time point as the reference image of another slice. To improve the robustness of the reference image selection approach, a reference time point during air inhalation could be selected for use across all slices.

The inhalation of pure oxygen can induce physiological changes, which may contribute to the observed MR signal, such as changes to the pulmonary blood volume or blood vessel diameter. Some studies report no effect of hyperoxia on pulmonary blood volume,^32^, ^33^ whereas a recent OE‐MRI study at 0.55 T by Wieslander et al.^34^ concluded that the pulmonary blood volume was altered by hyperoxia‐induced vasodilation. Wieslander et al. measured a hyperoxia‐induced 2% increase in lung proton density, which was attributed to the generation of a small but quantifiable contribution to the T_1_‐weighted OE‐MRI signal. Hyperoxia‐induced vasodilation could alter our OE‐MRI signal measurement in two main ways. Firstly, an increase in the pulmonary blood volume would increase the proton density of the lung, consequently increasing the measured lung MR signal. The increased lung MR signal would reduce the amplitude of the negative lung PSE, which could incorrectly be attributed to a reduced oxygen‐enhancement response. Alternatively, T_2_^*^ could be altered by vasodilation and geometrical changes of the lung structure. Models by Weisskoff et al.^35^ and Boxerman et al.^36^ for susceptibility‐induced Δ$R_{2}^{*}$ by vasculature in the brain suggest vasodilation causes T_2_^*^ to shorten. Vasodilation‐induced T_2_^*^ shortening would augment the susceptibility related T_2_^*^ shortening that already occurs due to the inhalation of pure oxygen. Greater T_2_^*^ shortening would produce a more negative PSE, reinforcing the oxygen‐induced signal changes within the lung tissue. The magnitude of such T_2_^*^ shortening is, however, difficult to predict for the greater magnetic susceptibility gradients within the lung relative to those used in the cerebral hemodynamics models by Weisskoff et al. and Boxerman et al.

Physiological changes such as absorption atelectasis may be induced by the inhalation of pure oxygen.^37^ The local alveolar shrinkage that occurs due to absorption atelectasis is likely to affect the measured MR signal. Alveolar shrinkage would decrease the volume of gaseous oxygen in the lung, potentially reducing the gas–tissue magnetic susceptibility gradients present and consequently increasing the local T_2_^*^. An increase in the local T_2_^*^ would increase the measured MR signal and contribute a positive PSE. However, the greater surface area‐to‐volume ratio of the smaller alveoli may reduce the local T_2_^*^ and provide a negative PSE. Alveolar shrinkage would also cause the local lung proton density to increase. As a result of the greater lung proton density, the measured MR signal would increase, contributing a positive PSE. Alteration of proton density due to absorption atelectasis may have contributed to the 2% increase in proton density reported by Wieslander et al. Through alteration of the local lung T_2_^*^ and proton density, absorption atelectasis induced by the inhalation of 100% oxygen may affect the measured MR signal and PSE.

### 5.4 Variation of PSE with slice position

For subjects in a supine position, the posterior lung slices may present a greater blood vessel density and increased ventilation due to gravity. A greater blood vessel density is likely to reduce the magnitude of the negative lung PSE due to partial volume effects from oxygenated blood vessels within the lung that produce a positive PSE (contrast mechanisms A and C in Figure 1). A greater blood vessel density may have given rise to the smaller magnitude of negative lung PSE_ICA_ measured in the posterior slices of echo 1 (Figure S9). For echo 2, a more negative PSE_ICA_ was measured in the posterior slices, suggesting that the trend of PSE_ICA_ in posterior slices was caused by a different source of contrast. Negative lung PSE occurs due to the presence of gaseous oxygen, which induces T_2_^*^‐shortening (contrast mechanism B in Figure 1). The increased ventilation of posterior slices could result in greater T_2_^*^‐shortening, which would act to increase the magnitude of the negative lung PSE_ICA_ measured at echo 2 in the posterior slices, as was observed.

Comparisons between the PSE measured in anterior and posterior slices were limited by the number of slices acquired and the resulting lung coverage. Due to SAR limitations, the dynamic OE‐MRI sequence acquired four slices covering 5.2 cm of the lung. In addition, the slice positioning was not designed to precisely assess trends of PSE_ICA_. As a result, variation in the slice positioning between participants may obscure the PSE_ICA_ trends. Further investigation into differences between the measured PSE of anterior and posterior slices may reveal differences in the contribution of ΔT_1_ and ΔT_2_^*^ contrast to the PSE with slice position. Such an investigation would require an increased coverage of lung volume using either a greater number of slices or a 3D acquisition.

## 6 CONCLUSION

The novel application of ICA to lung OE‐MRI enabled the separation of the ICA component relating to the oxygen‐induced signal change from confounding factors present in the lung, improving the accuracy of lung OE‐MRI analysis. At 3 T, the OE‐MRI analysis pipeline that we have developed was sensitive to, and enabled the resolution of, lung tissue from oxygenated blood. Our results demonstrated good scan–rescan and ICA pipeline repeatability, indicating robustness of the developed pipeline. We showed that the analysis pipeline was sensitive to smoking status, suggesting a likely sensitivity to pathology to be explored in future clinical studies.

## CONFLICT OF INTEREST

G.J.M. Parker is an employee of and holds ownership interest in Bioxydyn Limited. He also holds ownership interest in Queen Square Analytics Limited and Quantitative Imaging Limited. J.H. Naish and M. Tibiletti are employees of Bioxydyn Limited. No potential conflicts of interest were disclosed by the other authors.

## FUNDING INFORMATION

This work is supported by the Engineering & Physical Sciences Research Council (EPSRC)‐funded University College London (UCL) Centre for Doctoral Training in Medical Imaging, grant (EP/L016478/1), the Cancer Research UK National Cancer Imaging Translational Accelerator (NCITA) award 1519/A28682 (UCL) and C19221/A28683 (University of Manchester), and Innovate UK award 104629. J.R. McClelland acknowledges funding from Cancer Research UK (CRUK) Cambridge Institute via the Network Accelerator Award Grant (A21993) to the ART‐NET consortium and the Wellcome/EPSRC Centre for Interventional and Surgical Sciences (WEISS), grant (203145/Z/16/Z). This study represents independent research supported by the Manchester NIHR Biomedical Research Centre and by the National Institute for Health Research (NIHR) Biomedical Research Centre at The Royal Marsden NHS Foundation Trust and the Institute of Cancer Research, London.

## Supporting information


**FIGURE S1.** Diagram of the cyclic OE‐MRI gas delivery scheme involving three periods of 100% O_2_ inhalation. Gases were switched between medical air (21% O_2_) and 100% O_2_ every 1.5 min.
**FIGURE S2.** Example masks (light blue) overlaid on anatomical images. (A) lung mask: lung, excluding major vessels; (B) thoracic cavity mask: lung, heart, and major vessels.
**FIGURE S3.** Diagram to illustrate the application of ICA to the dual‐echo OE‐MRI data and the approach devised to identify the optimal oxygen‐enhancement ICA component.
**FIGURE S4.** Sequence‐specific MR simulations to predict the oxygen‐enhancements of lung tissue and oxygenated blood for (A) the change in signal (ΔS) and (B) the percentage signal enhancement (PSE).^2,3^ Solid vertical lines indicate the echo times used for the Philips Ingenia (TE_1,P_ and TE_2,P_) and Siemens MAGNETOM Vida (TE_1,S_ and TE_2,S_) scans.
Lung relaxation times used: T_1,air_ = 1281 ms and T_1,oxy_ = 1102 ms^4^; T_2,air_^*^ = 0.68 ms and T_2,oxy_^*^ = 0.62 ms^2^. Blood (oxygenated) relaxation times used: T_1,air_ = 1649 ms^5^ and T_1,oxy_ = 1354 ms^6^; T_2,air_^*^ = 59.4 ms and T_2,oxy_^*^ = 72.5 ms.^7^
For TE < 0.23 ms (TE = 0.23 ms indicated by a dotted vertical line) the signal simulation predicts a positive lung PSE due to the dominance of T_1_ effects, whereas for TE > 0.23 ms the simulation predicts a negative lung PSE due to the dominance of T_2_^*^ effects. Shown in (B), the PSE becomes more negative with increasing echo time.
**FIGURE S5.** The median lung PSE_ICA_ time series for the subjects presented in Figure 2. All echo 1 data for (A) non‐smoker participants and (B) current smoker participants, shown with a y‐axis range of −10% to 5% PSE.
**FIGURE S6.** The ICA components extracted from echo 1 of a non‐smoking participant, shown for the run of ICA in which the optimal OE ICA component was identified (22 components were used). The components are shown ordered by Spearman correlation value; the ordering metric value of each component is provided. The ordering approach successfully identified the OE ICA component as component 1. The OE ICA component displayed clear cyclic oxygen‐enhancement with signal changes occurring upon the switching of gases. The ICA components have an arbitrary scaling and undetermined sign.
**FIGURE S7.** The ICA components extracted from echo 2 of the same non‐smoking participant shown in Figure S6. The components presented are from the run of ICA in which the optimal OE ICA component was identified (23 components were used). The components are shown ordered by Spearman correlation value; the ordering metric value of each component is provided. The ordering approach successfully identified the OE ICA component as component 1. As for echo 1, the OE ICA component for echo 2 displayed clear cyclic oxygen‐enhancement with signal changes occurring upon the switching of gases. The ICA components have an arbitrary scaling and undetermined sign.
**FIGURE S8.** Frequency spectra of (A) the PSE_MRI_ time series and (B) the PSE_ICA_ time series shown in Figure 3. The frequency ranges associated with physiological motion and the OE‐MRI gas cycling are indicated on the spectra: respiratory frequencies, *f*_r_; aliased cardiac frequencies, *f*_c_; and gas cycling frequency, *f*_OE_ (also shaded in blue). The PSE_ICA_ spectrum contained a peak at the *f*_OE_ and minimal amplitudes at frequencies greater than *f*_OE_. In contrast, the PSE_MRI_ spectrum did not contain a sharp peak at *f*_OE_ and displayed substantial frequency content above *f*_OE_, particularly within *f*_r_ and *f*_c_.
**FIGURE S9.** Comparison between the median lung PSE_ICA_ measured in each of the four acquired slices for (A) echo 1 and (B) echo 2 of non‐smoker participants. For echo 1, the median lung PSE_ICA_ was less negative in posterior slices (slice 4 being most posterior). For echo 2, the median lung PSE_ICA_ was more negative in posterior slices. Table S4 presents a comparison of the median PSE_ICA_ between the slices of each echo using a sign test. The median lung PSE_ICA_ of slice 3 was significantly different to both slice 1 (*p* = 0.008) and slice 2 (*p* = 0.001) for echo 2.
**FIGURE S10.** The median lung PSE map value of each participant plotted against age for each echo of (A) the PSE_ICA_ data and (B) the PSE_MRI_ data.
**FIGURE S11.** Comparison of the median lung PSE map value of male (gray) and female (white) participants for each echo of (A) the PSE_ICA_ data and (B) the PSE_MRI_ data.
**FIGURE S12.** Bland–Altman plots of the IQR of the lung PSE_ICA_ for (A) the scan‐rescan repeatability and (B) the ICA pipeline repeatability. The solid black line indicates the bias and the dashed black lines indicate the limits of agreement. Bland–Altman analysis results are presented in full in Table S6.
**TABLE S1.** Details of (A) the non‐smoker and (B) the current smoker groups involved in the healthy participant study.
**TABLE S2.** Details of the free‐breathing dynamic lung OE‐MRI sequences implemented on (A) the Philips Ingenia scanner and (B) the Siemens MAGNETOM Vida scanner.
**TABLE S3.** Details of the NiftyReg^1^ parameters used to motion correct the dynamic images.
**TABLE S4.** Comparison of the median lung PSE_ICA_ between the four acquired slices of the non‐smoker participants for (A) echo 1 and (B) echo 2. A paired test (sign test) was used to make the comparisons. The Bonferroni correction for multiple comparisons was applied; *p* < 0.008 was considered significant. The median lung PSE_ICA_ measured in slice 3 was significantly different to that of slice 1 (*p* = 0.008) and slice 2 (*p* = 0.001) for echo 2.
**TABLE S5.** Variable coefficients and their significance in the multivariable models generated to adjust for the confounds of age and gender on the comparison between the median lung PSE of non‐smoker and current smoker participants. The median lung PSE of participants are plotted against age and gender in Figures S10 and S11, respectively.
Separate multiple regression models were created for: (A) echo 1 PSE_ICA_ (R^2^ = 0.474); (B) echo 2 PSE_ICA_ (R^2^ = 0.579); (C) echo 1 PSE_MRI_ (R^2^ = 0.301); (D) echo 2 PSE_MRI_ (R^2^ = 0.346). Current smoking status remained significant in the PSE_ICA_ data for both echoes when adjusted for age and gender. Current smoking status was not significant for the PSE_MRI_ data, both with and without adjustment for age and gender. Age was significant in the adjusted model for both echoes of the PSE_MRI_ data.
**TABLE S6.** Summary statistics from the analysis of the median lung PSE_ICA_ of: (A) the scan‐rescan repeatability; (B) the ICA pipeline repeatability; and (C) the multi‐site reproducibility. Also included are the statistics from the analysis of the IQR of the lung PSE_ICA_ of: (D) the scan‐rescan repeatability; and (E) the ICA pipeline repeatability. The bias, limits of agreement (LoA), repeatability coefficient (RC), and intra‐class correlation coefficient (ICC), were calculated for the scan‐rescan repeatability (A and D) and ICA repeatability (B and E). Only the bias and LoA were calculated for the reproducibility study (C) as the measurement conditions were not identical for the two scans – longer echo times were implemented on the Siemens MAGNETOM Vida than the Philips Ingenia.
