# Supplementary material for: Independent component analysis (ICA) applied to dynamic oxygen‐enhanced MRI (OE‐MRI) for robust functional lung imaging at 3 T
Source: Magn Reson Med. 2023 Nov 20;91(3):955–71. doi: 10.1002/mrm.29912 (PMC10952250; doi:10.1002/mrm.29912)
Supplement: Supplementary file 1 — FIGURE S1. Diagram of the cyclic OE‐MRI gas delivery scheme involving three periods of 100% O2 inhalation. Gases were switched between medical air (21% O2) and 100% O2 every 1.5 min. FIGURE S2. Example masks (light blue) overlaid on anatomical images. (A) lung mask: lung, excluding major vessels; (B) thoracic cavity mask: lung, heart, and major vessels. FIGURE S3. Diagram to illustrate the application of ICA to the dual‐echo OE‐MRI data and the approach devised to identify the optimal oxygen‐enhancement ICA component. FIGURE S4. Sequence‐specific MR simulations to predict the oxygen‐enhancements of lung tissue and oxygenated blood for (A) the change in signal (ΔS) and (B) the percentage signal enhancement (PSE).2,3 Solid vertical lines indicate the echo times used for the Philips Ingenia (TE1,P and TE2,P) and Siemens MAGNETOM Vida (TE1,S and TE2,S) scans. Lung relaxation times used: T1,air = 1281 ms and T1,oxy = 1102 ms4; T2,air * = 0.68 ms and T2,oxy * = 0.62 ms2. Blood (oxygenated) relaxation times used: T1,air = 1649 ms5 and T1,oxy = 1354 ms6; T2,air * = 59.4 ms and T2,oxy * = 72.5 ms.7 For TE < 0.23 ms (TE = 0.23 ms indicated by a dotted vertical line) the signal simulation predicts a positive lung PSE due to the dominance of T1 effects, whereas for TE > 0.23 ms the simulation predicts a negative lung PSE due to the dominance of T2 * effects. Shown in (B), the PSE becomes more negative with increasing echo time. FIGURE S5. The median lung PSEICA time series for the subjects presented in Figure 2. All echo 1 data for (A) non‐smoker participants and (B) current smoker participants, shown with a y‐axis range of −10% to 5% PSE. FIGURE S6. The ICA components extracted from echo 1 of a non‐smoking participant, shown for the run of ICA in which the optimal OE ICA component was identified (22 components were used). The components are shown ordered by Spearman correlation value; the ordering metric value of each component is provided. The ordering approach successfully i [file MRM-91-955-s001.docx]

**Supporting Information**

Supporting Information Table S1: Details of (A) the non-smoker and (B) the current smoker groups involved in the healthy participant study.

|  | | **(A)** | **(B)** |
| --- | --- | --- | --- |
| Participants | Total | 18 | 5 |
|  | Male | 8 | 5 |
|  | Female | 10 | 0 |
| Age (years) | Mean | 33 | 39 |
|  | Range | 22-54 | 27-55 |
| Smoking status | Currently smoke regularly | 0 | 5 |
| Pack years | Mean | 1.2 | 6.4 |
|  | Range | 0-8 | 1-14 |

Supporting Information Table S2: Details of the free-breathing dynamic lung OE-MRI sequences implemented on (A) the Philips Ingenia scanner and (B) the Siemens MAGNETOM Vida scanner.

|  | **(A)** | **(B)** |
| --- | --- | --- |
| Manufacturer | Philips | Siemens |
| Model | Ingenia | MAGNETOM Vida |
| Location | London | Manchester |
| Field strength (T) | 3 | 2.9 |
| RF coil used | 32-channel torso coil in combination with the posterior coil | 18 channel body coil in combination with the 32-channel spine coil |
| Max. gradient strength (mT/m) | 45 | 45 |
| Max. slew rate (T/m/s) | 200 | 200 |
| TR (ms) | 16 | 16 |
| Echoes | full | half |
| Minimum achievable TE_1_ (ms) | 0.71 | 0.81 |
| Minimum achievable TE_2_ (ms) | 1.2 | 1.51 |
| FOV (mm x mm) | 450 x 450 | 450 x 450 |
| No of slices | 4 | 4 |
| Slice thickness (mm) | 10 | 10 |
| Gap (mm) | 4 | 4 |
| Acquired matrix | 96 x 96 | 96 x 96 |
| Orientation | Coronal | Coronal |
| Pixel size (mm x mm) | 4.7 x 4.7 | 4.7 x 4.7 |
| Flip Angle (°) | 5 | 5 |
| Bandwidth (Hz/Px) | 4488 | 2000 |
| Parallel Imaging | N | N |
| NSA | 1 | 1 |
| Time resolution (s) | 1.54 | 1.54 |
| Number of dynamics | 420 | 420 |

Supporting Information Table S3: Details of the NiftyReg^1^ parameters used to motion correct the dynamic images.

| Multi-resolution levels | 3 |
| --- | --- |
| Control point grid spacing | 3 voxels, 14.1 mm final spacing |
| Similarity metric | Locally normalized cross-correlation |
| Penalty terms and weights | Bending energy, 0.005; linear elastic energy, 0.01 |
| Transformation parameterization | Stationary velocity field |
| Maximum number of iterations per resolution level | 600, 300, 150 |

Supporting Information Table S4: Comparison of the median lung PSE_ICA_ between the four acquired slices of the non-smoker participants for (A) echo 1 and (B) echo 2. A paired test (sign test) was used to make the comparisons. The Bonferroni correction for multiple comparisons was applied; *p* < 0.008 was considered significant. The median lung PSE_ICA_ measured in slice 3 was significantly different to that of slice 1 (*p* = 0.008) and slice 2 (*p* = 0.001) for echo 2.

| **(A)** | *p*-value | | | |
| --- | --- | --- | --- | --- |
|  | Slice 1 | Slice 2 | Slice 3 | Slice 4 |
| Slice 1 (anterior) |  | 0.238 | 0.815 | 0.031 |
| Slice 2 | 0.238 |  | 0.481 | 0.096 |
| Slice 3 | 0.815 | 0.481 |  | 0.238 |
| Slice 4 (posterior) | 0.031 | 0.096 | 0.238 |  |
| **(B)** | *p*-value | | | |
|  | Slice 1 | Slice 2 | Slice 3 | Slice 4 |
| Slice 1 (anterior) |  | 0.096 | 0.008 | 0.031 |
| Slice 2 | 0.096 |  | 0.001 | 0.096 |
| Slice 3 | 0.008 | 0.001 |  | 0.815 |
| Slice 4 (posterior) | 0.031 | 0.096 | 0.815 |  |

Supporting Information Table S5: Variable coefficients and their significance in the multivariable models generated to adjust for the confounds of age and gender on the comparison between the median lung PSE of non-smoker and current smoker participants. The median lung PSE of participants are plotted against age and gender in Supporting Information Figures S10 and S11, respectively.

Separate multiple regression models were created for: (A) echo 1 PSE_ICA_ (R^2^ = 0.474); (B) echo 2 PSE_ICA_ (R^2^ = 0.579); (C) echo 1 PSE_MRI_ (R^2^ = 0.301); (D) echo 2 PSE_MRI_ (R^2^ = 0.346). Current smoking status remained significant in the PSE_ICA_ data for both echoes when adjusted for age and gender. Current smoking status was not significant for the PSE_MRI_ data, both with and without adjustment for age and gender. Age was significant in the adjusted model for both echoes of the PSE_MRI_ data.

|  | **Variable** | **Standardized coefficient**  **[95% confidence interval]** | | **Unstandardized coefficient**  **[95% confidence interval]** | | **Significance** |
| --- | --- | --- | --- | --- | --- | --- |
| **(A)** | Current smoking status | 0.409 | [0.000, 0.817] | 1.791 | [0.000, 3.582] | **0.050** |
|  | Age | 0.205 | [-0.157, 0.568] | 0.040 | [-0.030, 0.109] | 0.250 |
|  | Gender | 0.326 | [-0.072, 0.723] | 1.188 | [-0.261, 2.637] | 0.102 |
| **(B)** | Current smoking status | 0.404 | [0.037, 0.772] | 2.745 | [0.254, 5.236] | **0.033** |
|  | Age | 0.294 | [-0.032, 0.620] | 0.088 | [-0.009, 0.185] | 0.074 |
|  | Gender | 0.370 | [0.013, 0.727] | 2.090 | [0.074, 4.106] | **0.043** |
| **(C)** | Current smoking status | 0.100 | [-0.371, 0.571] | 1.199 | [-4.433, 6.830] | 0.661 |
|  | Age | 0.455 | [0.037, 0.873] | 0.239 | [0.020, 0.458] | **0.034** |
|  | Gender | 0.218 | [-0.240, 0.676] | 2.168 | [-2.390, 6.725] | 0.332 |
| **(D)** | Current smoking status | 0.133 | [-0.323, 0.589] | 1.848 | [-4.479, 8.174] | 0.548 |
|  | Age | 0.451 | [0.046, 0.855] | 0.275 | [0.028, 0.521] | **0.031** |
|  | Gender | 0.265 | [-0.178, 0.709] | 3.064 | [-2.056, 8.184] | 0.226 |

Supporting Information Table S6: Summary statistics from the analysis of the median lung PSE_ICA_ of: (A) the scan-rescan repeatability; (B) the ICA pipeline repeatability; and (C) the multi-site reproducibility. Also included are the statistics from the analysis of the IQR of the lung PSE_ICA_ of: (D) the scan-rescan repeatability; and (E) the ICA pipeline repeatability. The bias, limits of agreement (LoA), repeatability coefficient (RC), and intra-class correlation coefficient (ICC), were calculated for the scan-rescan repeatability (A and D) and ICA repeatability (B and E). Only the bias and LoA were calculated for the reproducibility study (C) as the measurement conditions were not identical for the two scans - longer echo times were implemented on the Siemens MAGNETOM Vida than the Philips Ingenia.

|  |  | **Bias (PSE_ICA_ %)** | **Limits of agreement (PSE­_ICA_ %)** | **Repeatability coefficient (%)** | **ICC** |
| --- | --- | --- | --- | --- | --- |
| **(A)** | Echo 1 | -0.085 | [-2.257, 2.088] | 1.291 | 0.807 |
|  | Echo 2 | -0.023 | [-2.218, 2.172] | 1.512 | 0.907 |
| **(B)** | Echo 1 | -0.075 | [-1.502, 1.352] | 1.008 | 0.926 |
|  | Echo 2 | -0.240 | [-1.704, 1.223] | 1.152 | 0.958 |
| **(C)** | Echo 1 | 2.853 | [-1.992, 7.699] |  |  |
|  | Echo 2 | -4.095 | [-7.180, -1.010] |  |  |
| **(D)** | Echo 1 | 0.558 | [-1.043, 2.160] | 1.461 | 0.669 |
|  | Echo 2 | 0.685 | [-2.473, 3.800] | 2.189 | 0.525 |
| **(E)** | Echo 1 | -0.009 | [-0.602, 0.584] | 0.402 | 0.971 |
|  | Echo 2 | 0.156 | [-0.872, 1.184] | 0.783 | 0.942 |

Supporting Information Figure S1: Diagram of the cyclic OE-MRI gas delivery scheme involving three periods of 100% O_2_ inhalation. Gases were switched between medical air (21% O_2_) and 100% O_2_ every 1.5 minutes.


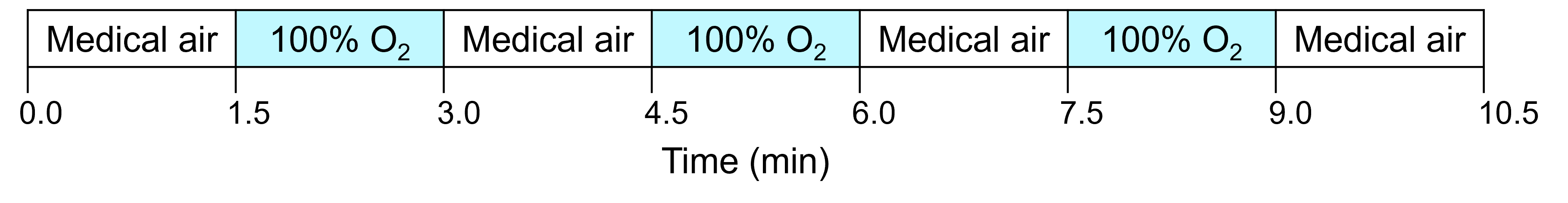


Supporting Information Figure S2: Example masks (light blue) overlaid on anatomical images. (A) lung mask: lung, excluding major vessels; (B) thoracic cavity mask: lung, heart, and major vessels.


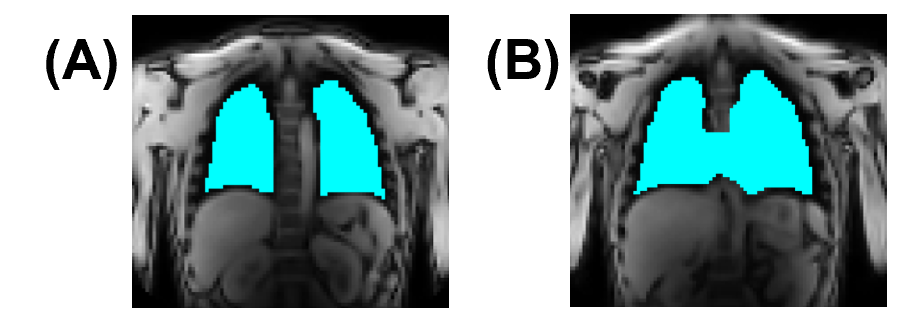


Supporting Information Figure S3: Diagram to illustrate the application of ICA to the dual-echo OE-MRI data and the approach devised to identify the optimal oxygen-enhancement ICA component.


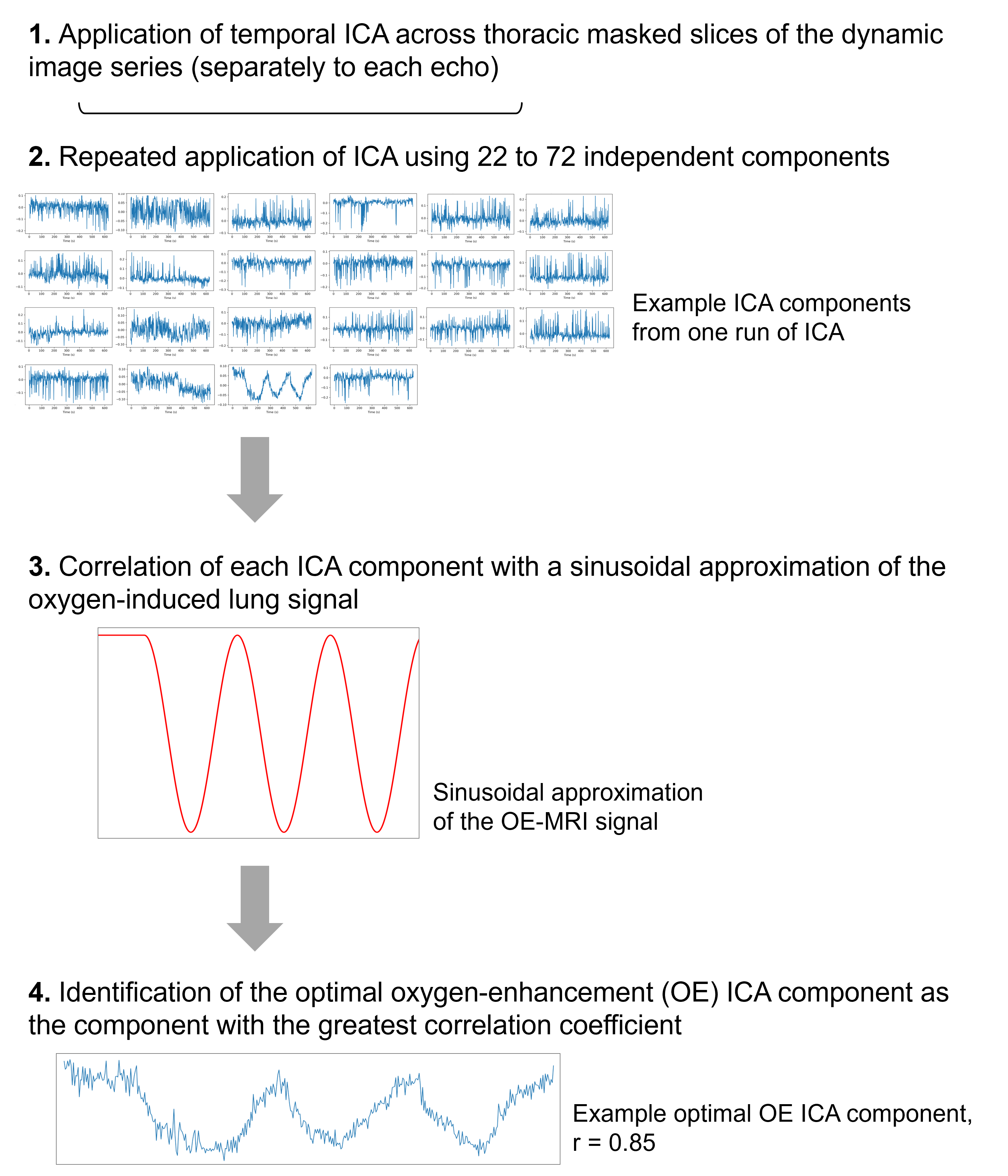


Supporting Information Figure S4: Sequence-specific MR simulations to predict the oxygen-enhancements of lung tissue and oxygenated blood for (A) the change in signal (ΔS) and (B) the percentage signal enhancement (PSE)^2,3^. Solid vertical lines indicate the echo times used for the Philips Ingenia (TE_1,P_ and TE_2,P_) and Siemens MAGNETOM Vida (TE_1,S_ and TE_2,S_) scans.

Lung relaxation times used: T_1,air_ = 1281 ms and T_1,oxy_ = 1102 ms^4^; T_2,air_^*^ = 0.68 ms and T_2,oxy_^*^ = 0.62 ms^2^. Blood (oxygenated) relaxation times used: T_1,air_ = 1649 ms^5^ and T_1,oxy_ = 1354 ms^6^; T_2,air_^*^ = 59.4 ms and T_2,oxy_^*^ = 72.5 ms^7^.

For TE < 0.23 ms (TE = 0.23 ms indicated by a dotted vertical line) the signal simulation predicts a positive lung PSE due to the dominance of T_1_ effects, whereas for TE > 0.23 ms the simulation predicts a negative lung PSE due to the dominance of T_2_^*^ effects. Shown in (B), the PSE becomes more negative with increasing echo time.


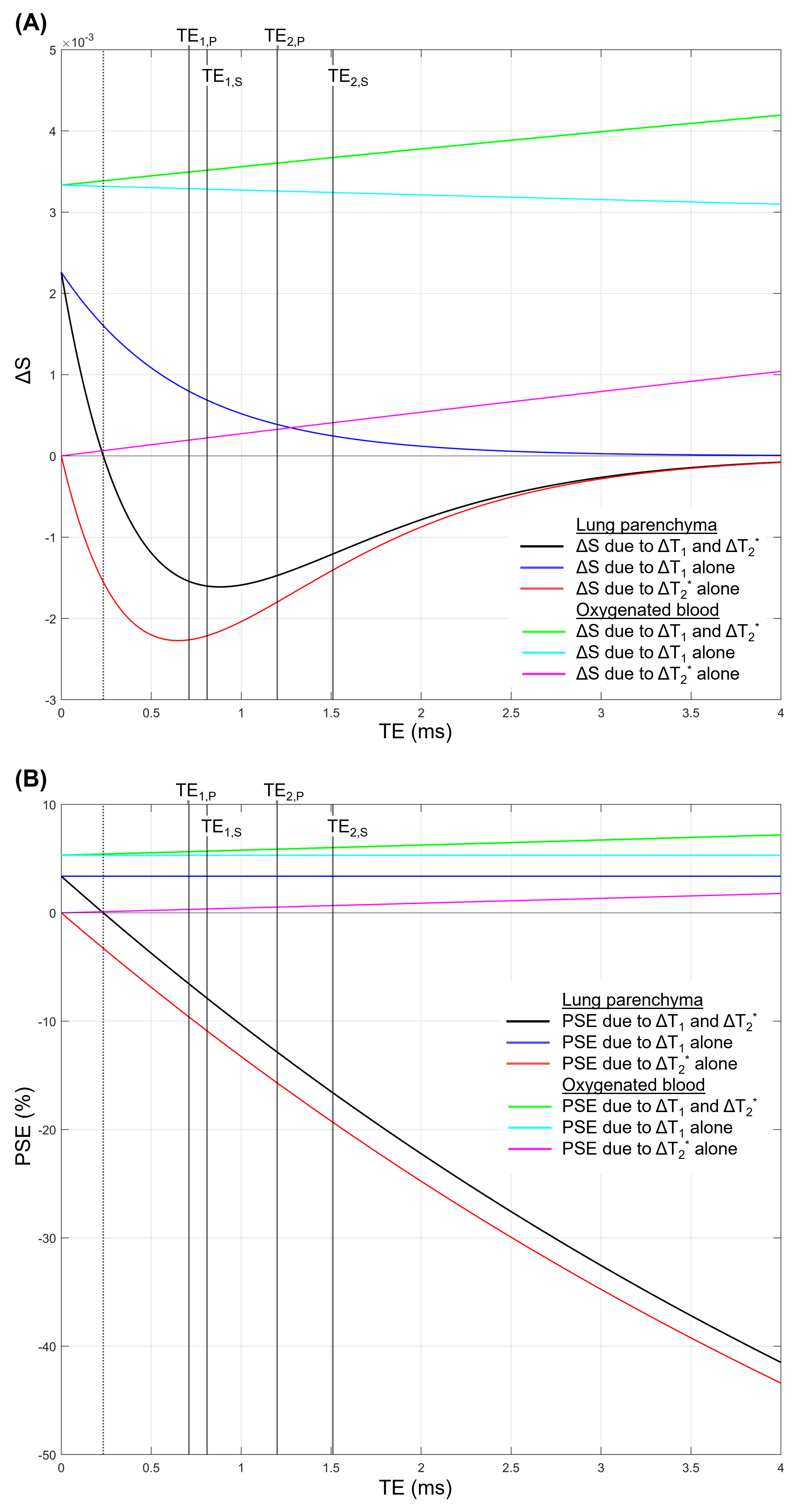


Supporting Information Figure S5: The median lung PSE_ICA_ time series for the subjects presented in Figure 2. All echo 1 data for (A) non-smoker participants and (B) current smoker participants, shown with a y-axis range of -10% to 5% PSE.


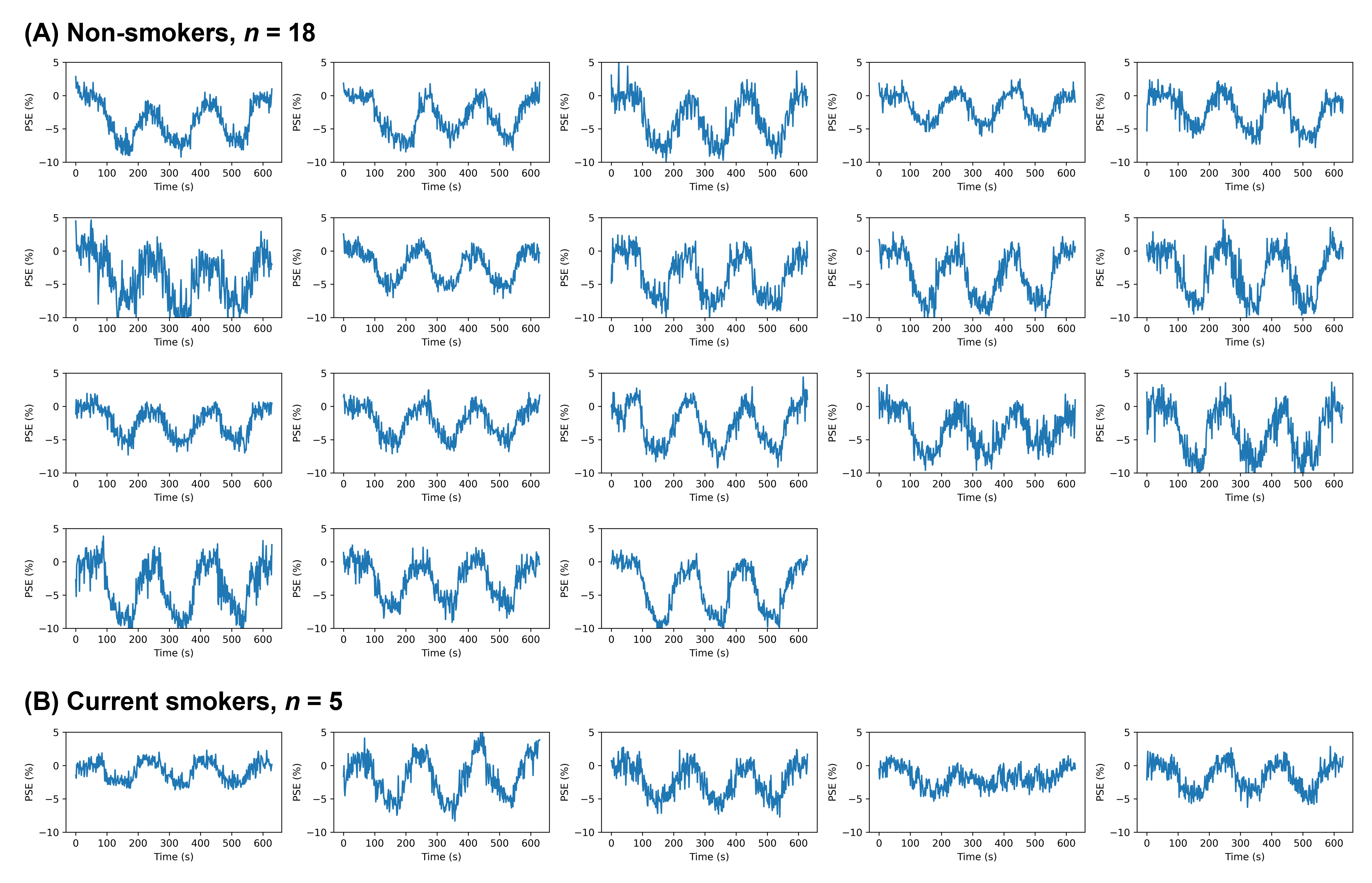


Supporting Information Figure S6: The ICA components extracted from echo 1 of a non-smoking participant, shown for the run of ICA in which the optimal OE ICA component was identified (22 components were used). The components are shown ordered by Spearman correlation value; the ordering metric value of each component is provided. The ordering approach successfully identified the OE ICA component as component 1. The OE ICA component displayed clear cyclic oxygen-enhancement with signal changes occurring upon the switching of gases. The ICA components have an arbitrary scaling and undetermined sign.


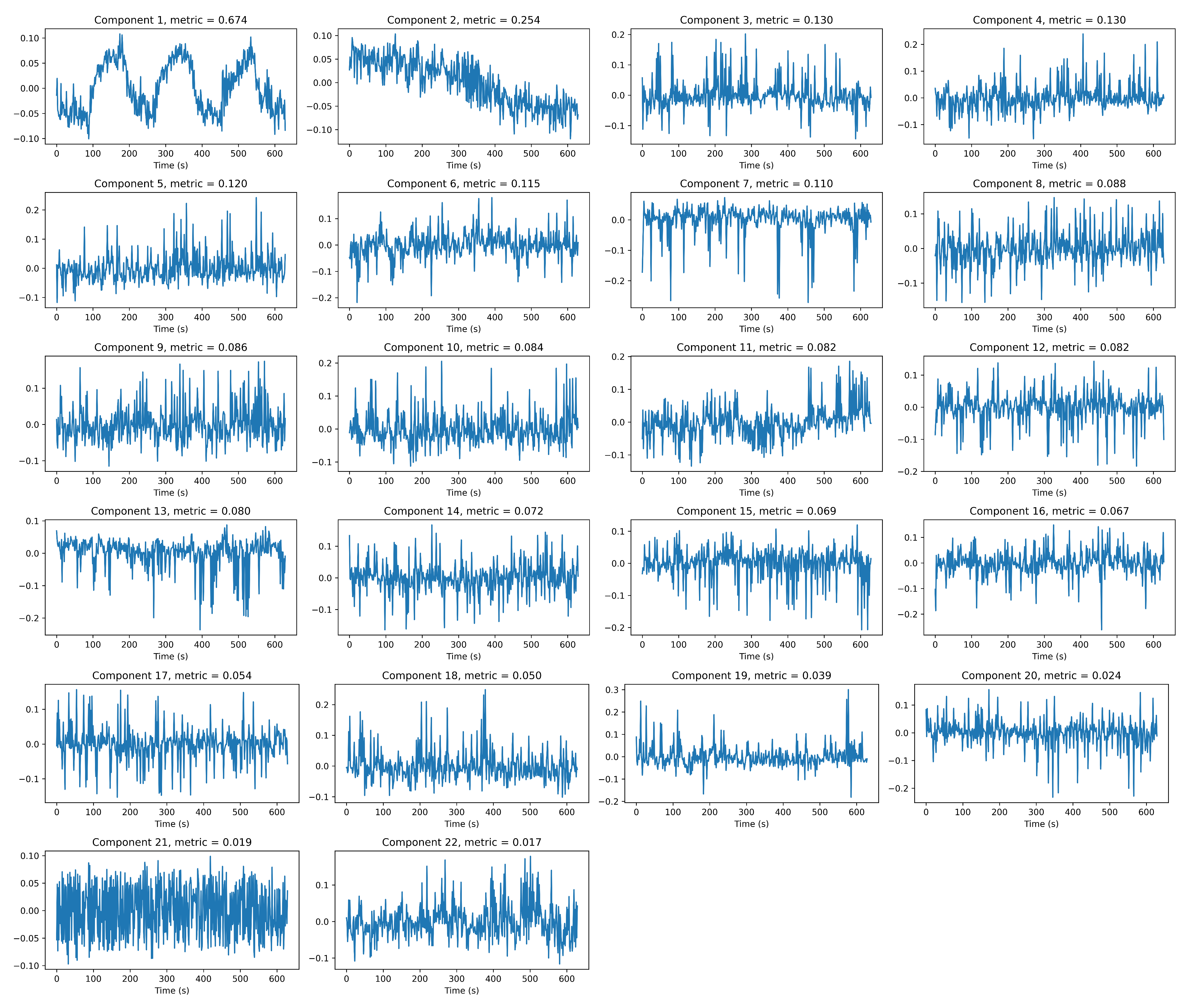


Supporting Information Figure S7: The ICA components extracted from echo 2 of the same non-smoking participant shown in Supporting Information Figure S6. The components presented are from the run of ICA in which the optimal OE ICA component was identified (23 components were used). The components are shown ordered by Spearman correlation value; the ordering metric value of each component is provided. The ordering approach successfully identified the OE ICA component as component 1. As for echo 1, the OE ICA component for echo 2 displayed clear cyclic oxygen-enhancement with signal changes occurring upon the switching of gases. The ICA components have an arbitrary scaling and undetermined sign.


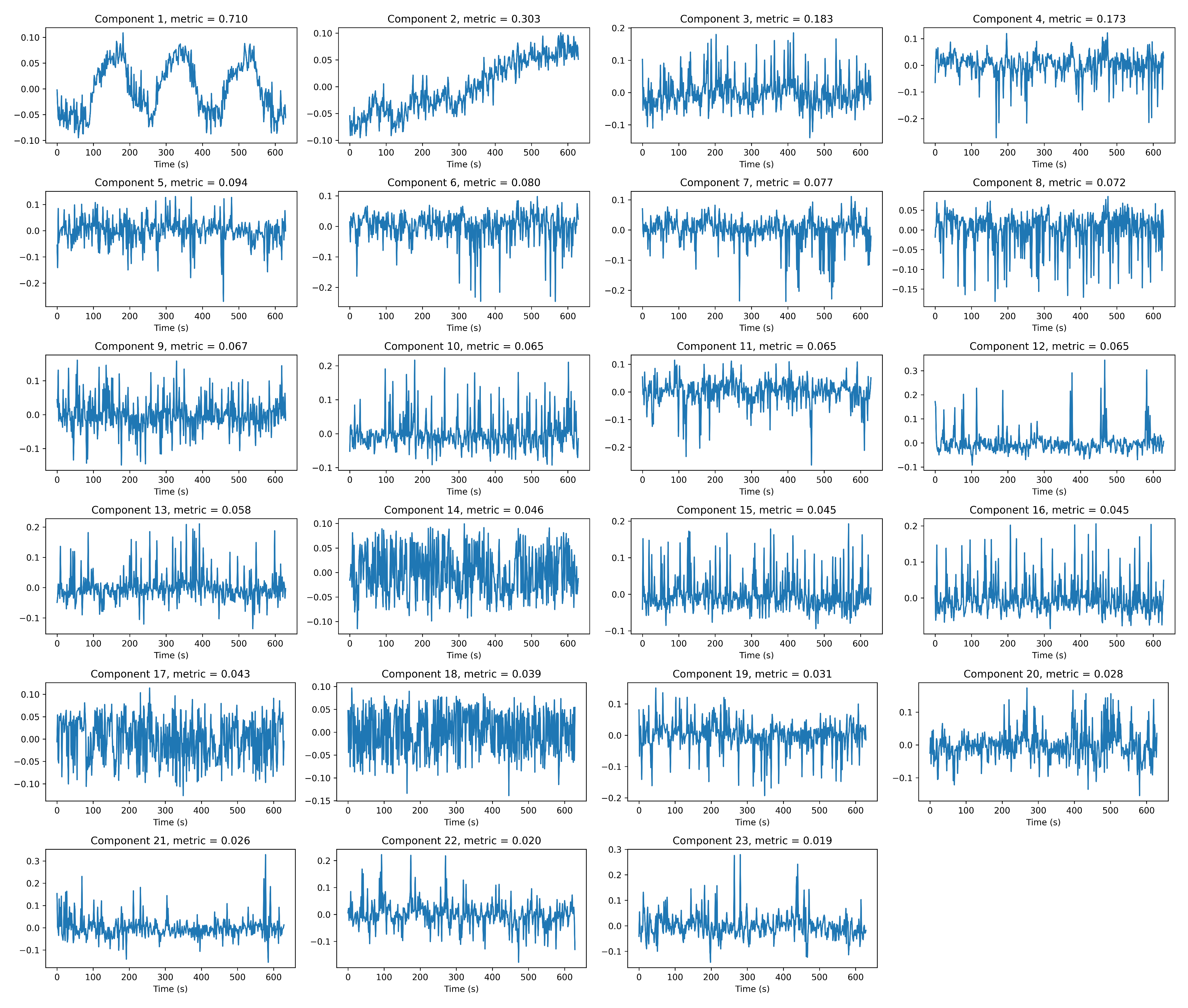


Supporting Information Figure S8: Frequency spectra of (A) the PSE_MRI_ time series and (B) the PSE_ICA_ time series shown in Figure 3. The frequency ranges associated with physiological motion and the OE-MRI gas cycling are indicated on the spectra: respiratory frequencies, *f*_r_; aliased cardiac frequencies, *f*_c_; and gas cycling frequency, *f*_OE_ (also shaded in blue). The PSE_ICA_ spectrum contained a peak at the *f*_OE_ and minimal amplitudes at frequencies greater than *f*_OE_. In contrast, the PSE_MRI_ spectrum did not contain a sharp peak at *f*_OE_ and displayed substantial frequency content above *f*_OE_, particularly within *f*_r_ and *f*_c_.


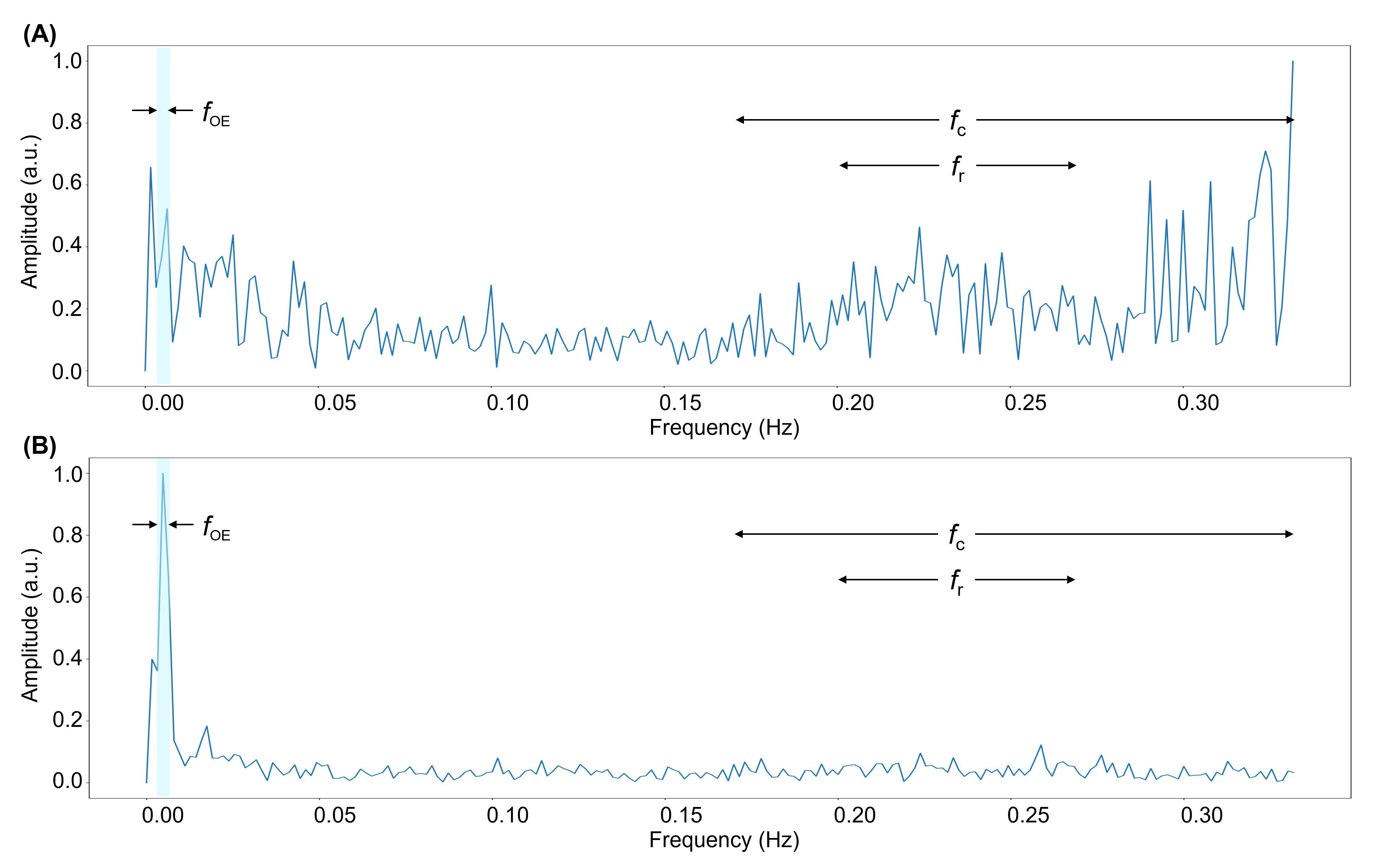


Supporting Information Figure S9: Comparison between the median lung PSE_ICA_ measured in each of the four acquired slices for (A) echo 1 and (B) echo 2 of non-smoker participants. For echo 1, the median lung PSE_ICA_ was less negative in posterior slices (slice 4 being most posterior). For echo 2, the median lung PSE_ICA_ was more negative in posterior slices. Supporting Information Table S4 presents a comparison of the median PSE_ICA_ between the slices of each echo using a sign test. The median lung PSE_ICA_ of slice 3 was significantly different to both slice 1 (*p* = 0.008) and slice 2 (*p* = 0.001) for echo 2.


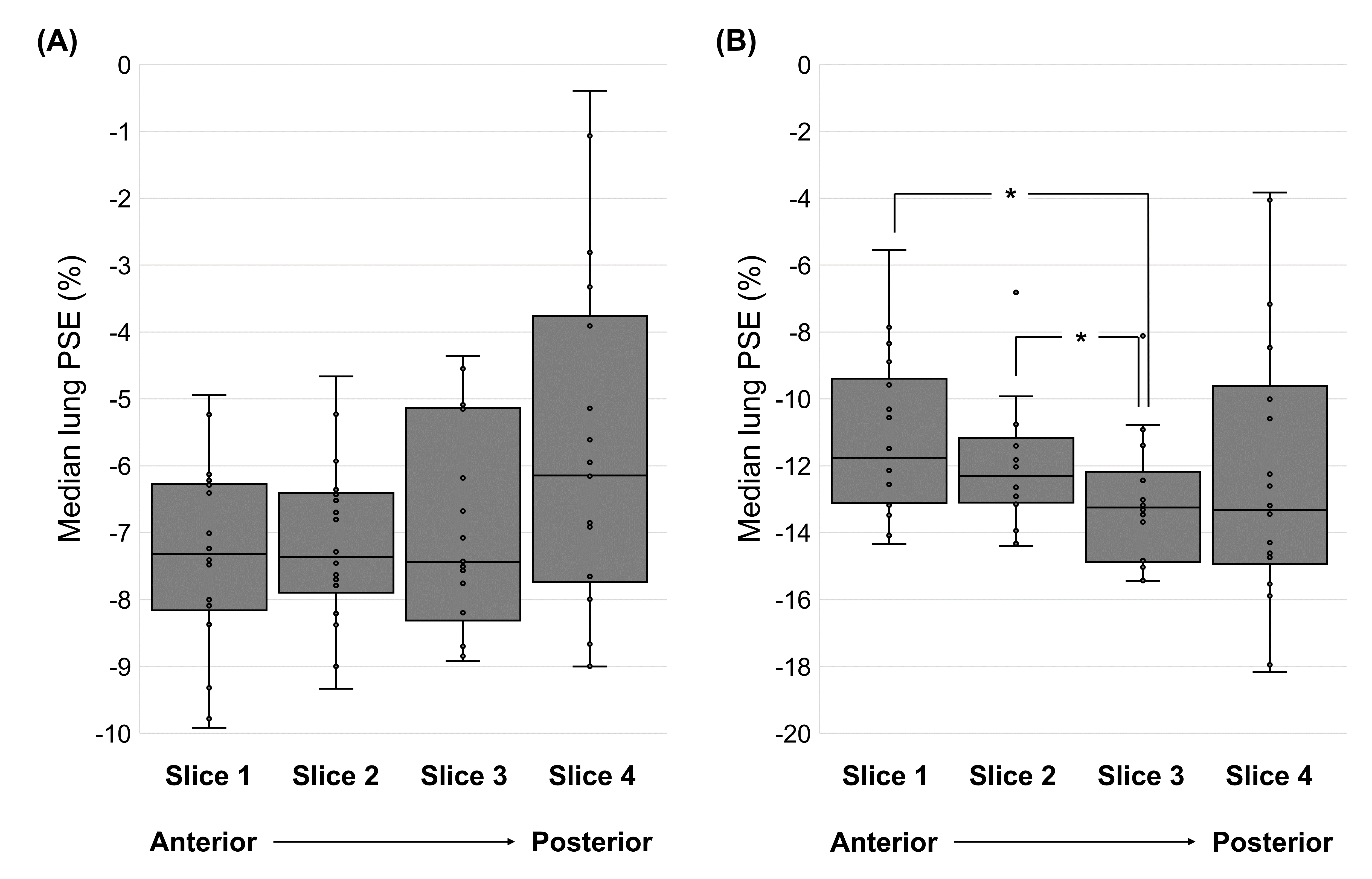


Supporting Information Figure S10: The median lung PSE map value of each participant plotted against age for each echo of (A) the PSE_ICA_ data and (B) the PSE_MRI_ data.


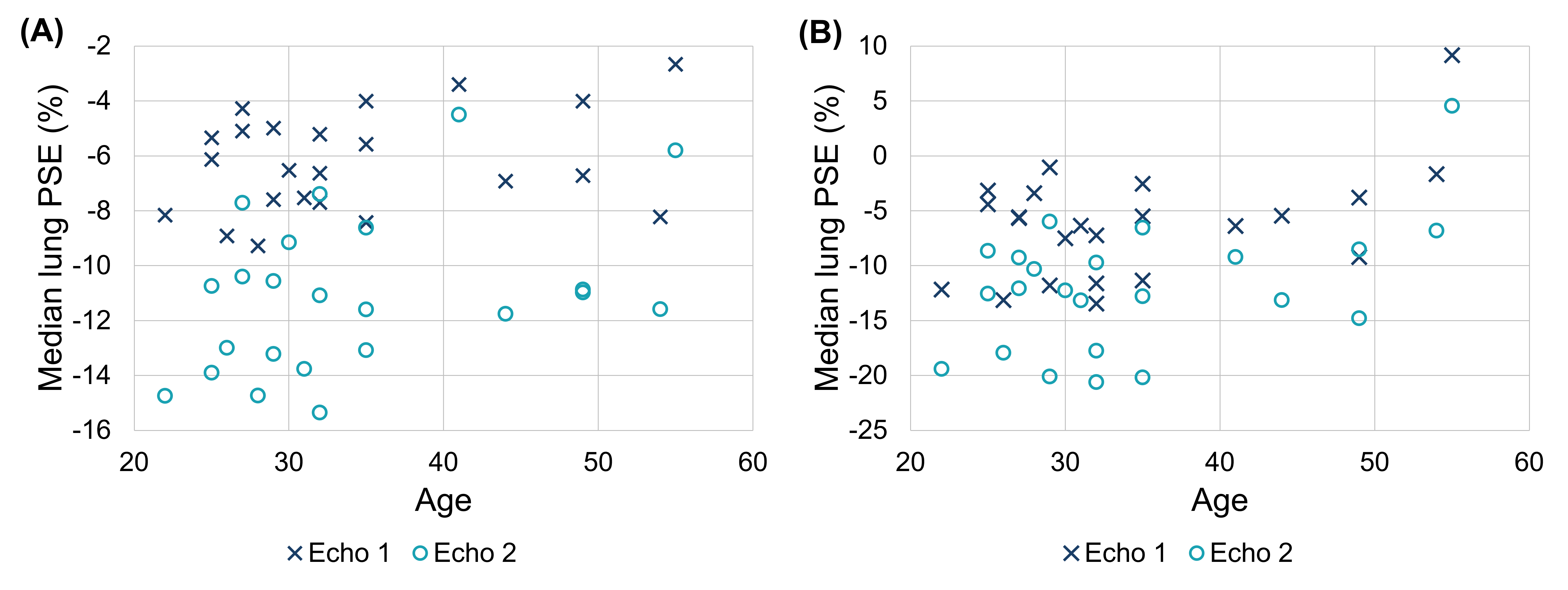


Supporting Information Figure S11: Comparison of the median lung PSE map value of male (gray) and female (white) participants for each echo of (A) the PSE_ICA_ data and (B) the PSE_MRI_ data.


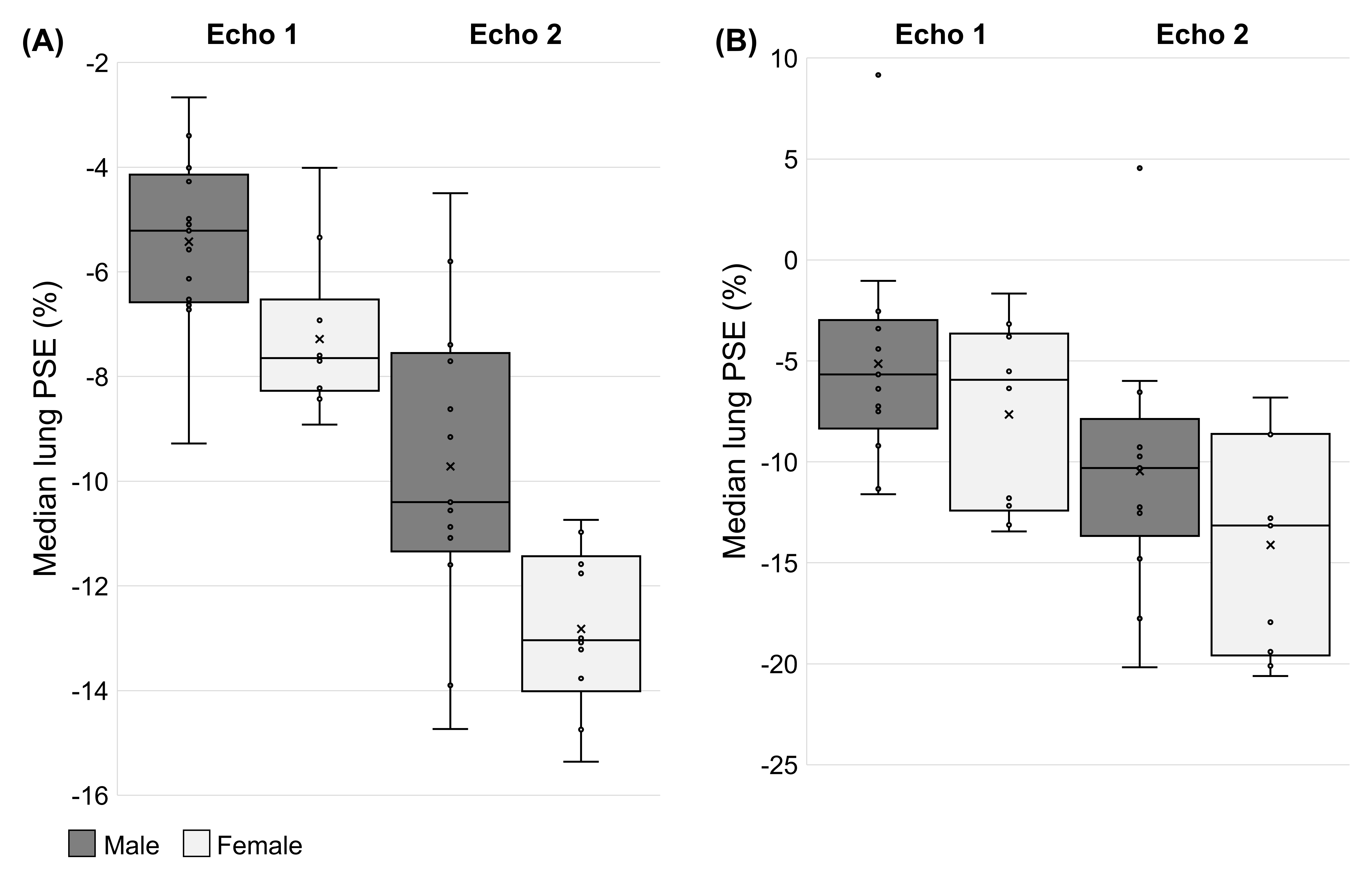


Supporting Information Figure S12: Bland-Altman plots of the IQR of the lung PSE_ICA_ for (A) the scan-rescan repeatability and (B) the ICA pipeline repeatability. The solid black line indicates the bias and the dashed black lines indicate the limits of agreement. Bland-Altman analysis results are presented in full in Supporting Information Table S6.


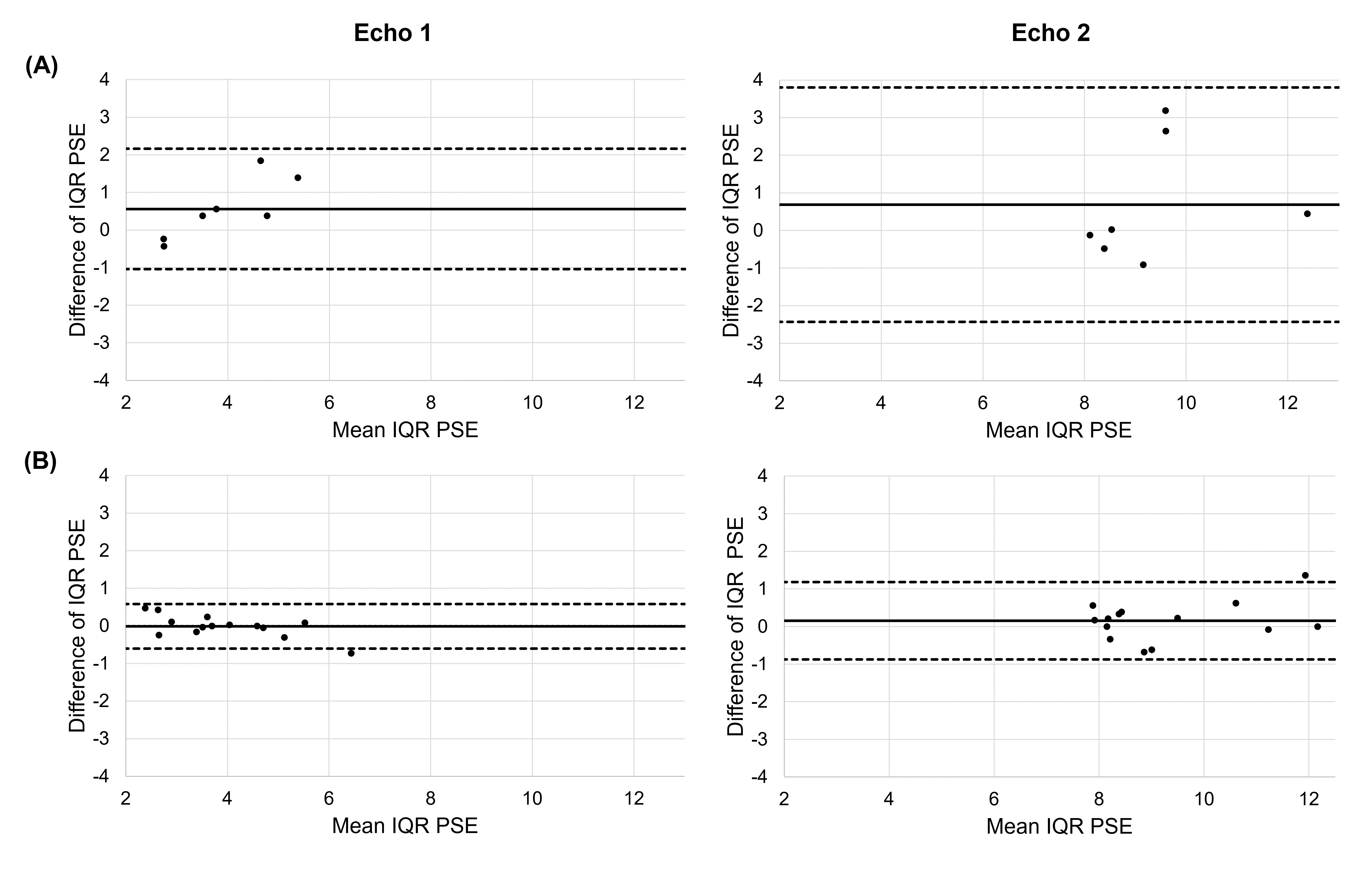


**References**

1. Modat M, Ridgway GR, Taylor ZA, Lehmann M, Barnes J, Hawkes DJ, et al. Fast free-form deformation using graphics processing units. *Comput Methods Programs Biomed*. 2010;98(3):278-284. doi:10.1016/j.cmpb.2009.09.002

2. Kim M, Naish JH, Needleman SH, Tibiletti M, Taylor Y, O’Connor JPB, et al. Dynamic oxygen-enhanced MRI of the lung at 3 T: feasibility, repeatability and reproducibility. *bioRxiv*. Published online January 1, 2023:2023.04.09.536144. doi:10.1101/2023.04.09.536144

3. Kruger SJ, Fain SB, Johnson KM, Cadman R V, Nagle SK. Oxygen-enhanced 3D radial ultrashort echo time magnetic resonance imaging in the healthy human lung. *NMR Biomed*. 2014;27(12):1535-1541. doi:10.1002/nbm.3158

4. Dietrich O, Raya J, Fasol U, Peller M, Reiser M, Schoenberg S. Oxygen-enhanced MRI of the lung at 3 Tesla: Feasibility and T1 relaxation times. In: *Proc. Intl. Soc. Mag. Reson. Med. 14*. ; 2006:1307.

5. Zhang X, Petersen ET, Ghariq E, De Vis JB, Webb AG, Teeuwisse WM, et al. In vivo blood T1 measurements at 1.5 T, 3 T, and 7 T. *Magn Reson Med*. 2013;70(4):1082-1086. doi:https://doi.org/10.1002/mrm.24550

6. Bluemke E, Stride E, Bulte DP. A General Model to Calculate the Spin–Lattice Relaxation Rate (R1) of Blood, Accounting for Hematocrit, Oxygen Saturation, Oxygen Partial Pressure, and Magnetic Field Strength Under Hyperoxic Conditions. *J Magn Reson Imaging*. 2022;55(5):1428-1439. doi:https://doi.org/10.1002/jmri.27938

7. Zhao JM, Clingman CS, Närväinen MJ, Kauppinen RA, van Zijl PCM. Oxygenation and hematocrit dependence of transverse relaxation rates of blood at 3T. *Magn Reson Med*. 2007;58(3):592-597. doi:https://doi.org/10.1002/mrm.21342
